# Modeling the solubility of light hydrocarbon gases and their mixture in brine with machine learning and equations of state

**DOI:** 10.1038/s41598-022-18983-2

**Published:** 2022-09-02

**Authors:** Mohammad-Reza Mohammadi, Fahimeh Hadavimoghaddam, Saeid Atashrouz, Ali Abedi, Abdolhossein Hemmati-Sarapardeh, Ahmad Mohaddespour

**Affiliations:** 1 Department of Petroleum Engineering, Shahid Bahonar University of Kerman, Kerman, Iran; 2 Key Laboratory of Continental Shale Hydrocarbon Accumulation and Efficient Development (Northeast Petroleum University), Ministry of Education, Northeast Petroleum University, Daqing, 163318 Heilongjiang China; 3 Institute of Unconventional Oil and Gas, Northeast Petroleum University, Daqing, 163318 China; 4 Department of Chemical Engineering, Amirkabir University of Technology (Tehran Polytechnic), Tehran, Iran; 5 College of Engineering and Technology, American University of the Middle East, Kuwait City, Kuwait; 6 College of Construction Engineering, Jilin University, Changchun, China; 7 Department of Chemical Engineering, McGill University, Montreal, QC H3A 0C5 Canada

**Keywords:** Energy science and technology, Engineering

## Abstract

Knowledge of the solubilities of hydrocarbon components of natural gas in pure water and aqueous electrolyte solutions is important in terms of engineering designs and environmental aspects. In the current work, six machine-learning algorithms, namely Random Forest, Extra Tree, adaptive boosting support vector regression (AdaBoost-SVR), Decision Tree, group method of data handling (GMDH), and genetic programming (GP) were proposed for estimating the solubility of pure and mixture of methane, ethane, propane, and *n*-butane gases in pure water and aqueous electrolyte systems. To this end, a huge database of hydrocarbon gases solubility (1836 experimental data points) was prepared over extensive ranges of operating temperature (273–637 K) and pressure (0.051–113.27 MPa). Two different approaches including eight and five inputs were adopted for modeling. Moreover, three famous equations of state (EOSs), namely Peng-Robinson (PR), Valderrama modification of the Patel–Teja (VPT), and Soave–Redlich–Kwong (SRK) were used in comparison with machine-learning models. The AdaBoost-SVR models developed with eight and five inputs outperform the other models proposed in this study, EOSs, and available intelligence models in predicting the solubility of mixtures or/and pure hydrocarbon gases in pure water and aqueous electrolyte systems up to high-pressure and high-temperature conditions having average absolute relative error values of 10.65% and 12.02%, respectively, along with determination coefficient of 0.9999. Among the EOSs, VPT, SRK, and PR were ranked in terms of good predictions, respectively. Also, the two mathematical correlations developed with GP and GMDH had satisfactory results and can provide accurate and quick estimates. According to sensitivity analysis, the temperature and pressure had the greatest effect on hydrocarbon gases’ solubility. Additionally, increasing the ionic strength of the solution and the pseudo-critical temperature of the gas mixture decreases the solubilities of hydrocarbon gases in aqueous electrolyte systems. Eventually, the Leverage approach has revealed the validity of the hydrocarbon solubility databank and the high credit of the AdaBoost-SVR models in estimating the solubilities of hydrocarbon gases in aqueous solutions.

## Introduction

One of the crucial theoretical and practical challenges in petroleum, chemical, and geochemical engineering is the solubilities of hydrocarbons, such as methane, ethane, propane, *n*-butane, or their mixtures, in pure water and aqueous electrolyte solutions. Achieving optimal conditions for gas and oil transportation, designing thermal separation processes, coal gasification, and hydrate formation require accurate information about the solubilities of hydrocarbon gases in different aqueous phases^1–5^. Natural gases coexist with aqueous solutions in petroleum reservoirs under the circumstances of high temperature and high pressure, which makes the solubilities of gases an important challenge for engineers. The water content of gases can undergo a phase alteration from vapor to gas hydrates, water condensate, and ice in the production and transportation of hydrocarbons. The condensed water phase in the compressor can damage impeller blades. Also, corrosion and pipeline blockage, as two serious flow assurance problems, can be caused by the formation of gas hydrates and/or ice throughout the production and transportation of hydrocarbons^1,6–8^. From an environmental point of view, gases solubility in water is a substantial problem because of the legislation and restrictions on the hydrocarbons contents in the water disposal^9^. In addition, leaking pipelines, underground oil storage tanks, and accidents on oil platforms and ships of the hydrocarbons’ transportation are responsible for oil spillage in water^10–12^.

Because of complex non-idealities from the strong H-bonding of water molecules, an accurate description of the phase behavior of these systems, utilizing theoretical methods is a challenging issue^13^. Accurate gas solubility data is essential to develop thermodynamic models for giving a qualified evaluation of the water content in the gases phase^9^. Therefore, the objective of thermodynamic calculations is the estimation of the compositions, content, and other equilibrium properties of the phases. Traditional equations of state (EOSs) are mainly applied to estimate thermodynamic and physical properties such as gas solubility. However, accurate estimates of gases solubility in various solvents by EOSs face serious problems such as iterative calculations, limited flexibility, and adjustable parameters at different temperatures and pressures. This makes the application of current conventional approaches, for example EOSs, unreliable and convinces researchers to seek better predictive techniques^14–19^.

The petroleum industry needs appropriate and precise knowledge of the correlation between operating conditions (i.e., pressure and temperature), vapor and liquid phases compositions, and the salinity of the aqueous phase for the systems containing aqueous electrolyte solutions and natural gas’ components. This knowledge can help design/optimize the operating condition for gas processing units and avoid/diagnose problems accompanying natural gas applications. Literature survey shows that there are many sets of experimental solubility data for various gas − liquid systems. Available experimental sources mainly present the solubility of pure hydrocarbon gases^2,4,20–22^, hydrocarbon gas mixtures^1,5,6,9,23–25^, and non-hydrocarbon gases (e.g., N_2_ and CO_2_)^26–30^ in water/brine systems. On the other hand, due to the difficulties encountered in measuring the low content of water of gases at low-temperature and high-pressure conditions, experimental data of water content of hydrocarbon and non-hydrocarbon gases are limited and scattered. However, Mohammadi et al.^1^ demonstrated that complexities associated with experimental measurement of the water content in natural gas could be eliminated by gas solubilities data, which provides an accurate estimate of water content^1^. Attempts to model the vapor–liquid phase equilibria of non-hydrocarbon and hydrocarbon gases and brine solutions have always been considered by researchers due to the limited number of measurements. The activity coefficient, Henry’s constant approach, and EOSs were widely used in thermodynamic models in order to gain information about the equilibrium conditions of non-hydrocarbon and hydrocarbon gases and pure water or aqueous electrolytes solutions^5,9,31–41^. Although Henry’s law can appropriately be utilized to estimate the solubilities, this approach has several drawbacks. For instance, this approach is correct for unique compounds at low concentrations under equilibria conditions with no chemical reactions for the aqueous phase. Also, it is appropriate for near-ideal or dilute solutions^42^. Moreover, at low temperatures, there is a limited count of Henry’s constants for the systems containing hydrocarbons-aqueous solutions^3^. On the other hand, the advantages such as lower count of parameters, the easiness of implementation, and computational efficiency make the use of EOSs widespread^2,4,9,43^. However, the accuracy of EOSs is highly dependent on the appropriation of empirical adjustments via incorporating the binary interaction parameters. Therefore, reliable sources of experimental data for the vapor–liquid equilibria of binary or even multi-component mixtures are essential to determine these parameters^23,44^. Hence, developing EOS for extensive applications such as calculations of natural gas’ solubility faces serious problems, and numerous EOSs developed so far are mostly attributed to limited systems. Due to the above discussions, in recent years, researchers have tried to provide accurate and reliable approaches to predict the solubilities of non-hydrocarbon and hydrocarbon gases in pure water and aqueous electrolyte systems. Literature survey shows that many intelligent models have been proposed to estimate the solubilities of non-hydrocarbon gases, especially CO_2_, in water and brine^45–50^. Regarding hydrocarbons solubility in pure water and brine, Safamirzaei et al.^51^ utilized a simple artificial neural network (ANN) with overall 101 solubility data points for modeling *n*-alkanes (*n*C1–*n*C6) solubilities in water. They showed that an ANN-based model could be an alternative to other methods such as EOSs with high accuracy^51^. Samani et al.^52^ proposed two hybrid models based on least-squares support vector machine and coupled simulated annealing algorithms for estimating the solubility of hydrocarbons (C1–C4) and non-hydrocarbon gases (CO_2_ and N_2_) in aqueous electrolyte systems. Regarding hydrocarbon gases, their database had 1175 solubility data points, and the average absolute error of their proposed model was 30.6%^52^. Nabipour et al.^53^ used a similar database including 1175 data points and an extreme learning machine algorithm to develop a model for predicting hydrocarbon gases (C1–C4) solubility in electrolyte solutions. The mean relative error of their model was 22.05%^53^. Although two relatively comprehensive intelligent models have been developed to predict the solubilities of hydrocarbon gases in aqueous electrolyte systems, the error of these models is slightly high. Also, due to the nature of the data-driven soft computing approaches, incorporating a larger number of data, various operating conditions, and adopting different modeling approaches may propel a comprehensive predictive tool for estimating the solubilities of light hydrocarbon gases and their mixture in water and aqueous electrolyte solutions. Furthermore, the development of easy-to-use mathematical correlations by advanced algorithms can simplify and accelerate the prediction of hydrocarbon gas solubilities in brine.

In this research, a huge database (1836 experimental data points) of hydrocarbon gases solubilities in pure water and aqueous electrolyte systems was accumulated from the literature. Next, for developing predictive tools, six robust machine learning algorithms viz., Random Forest, Extra Tree, adaptive boosting support vector regression (AdaBoost-SVR), Decision Tree, genetic programming (GP), and group method of data handling (GMDH) are implemented in this study by considering two different approaches. Additionally, three famous equations of state (EOSs) viz., Peng–Robinson (PR), Valderrama modification of the Patel–Teja (VPT), and Soave–Redlich–Kwong (SRK) are utilized in comparison with machine learning models. Furthermore, the performance of machine learning-based predictive tools and mathematical correlations is studied by employing various statistical and visual error analyses. Besides, a well-known sensitivity analysis, i.e., the relevancy factor, is identified the relative impact of input variables on hydrocarbon gases solubility in brine. Ultimately, the validity of the solubility databank, along with the application domain of the best-developed predictive tools in the present work, is examined by the Leverage mathematical method.

## Data acquisition

In this work, a large databank was collected on the basis of experimental solubility data of light hydrocarbon gases and their mixtures in water and aqueous electrolytes. This databank consists of 1836 data points that are 661 data points more than what is used in Samani et al.^52^ and Nabipour et al.^53^ works. Table 1 presents the details and references of experimental solubility data for hydrocarbon components of natural gas in pure water and aqueous electrolytes used in this survey. It should be noted that the collected laboratory data for the solubility of gases in pure water and brine is such that most of the solubility values were reported in two-phase conditions (a gaseous phase and an aqueous phase in equilibrium). This means that the temperature and pressure of the system were such that only two phases would exist in equilibrium. This is while there is a possibility of the formation of three phases at conditions of pressure higher than the critical pressure of components or low-temperature conditions. According to the Gibbs phase rule, degrees of freedom are the number of intensive properties that can be altered without varying the number of phases, or the number of components in any phase^54^. Hence, in some studies such as Amirijafari’s work^23^, for measuring hydrocarbon gas solubility in water under high-pressure conditions, the temperatures were selected such that only two phases (hydrocarbon gas mixture and the liquid water with hydrocarbons dissolved in it) would be present. Adopting this approach makes measuring gas solubilities easier and the obtained data more reliable. Although in some other studies^5,6^, in addition to measuring the solubility data in the two-phase state, the solubility values have been measured in the three-phase conditions, i.e. (three-phase equilibrium between the hydrate, the aqueous, and the vapor phase or three-phase equilibrium between water-rich liquid, hydrocarbon-rich liquid, and vapor phase). However, experimental measurements of solubilities in such a condition are challenging and could potentially generate unreliable laboratory data. For example, concentrations of light hydrocarbon gases in water are low, and moreover reaching the equilibrium states near and inside the gas hydrate formation region is a time-consuming process. However, the data collected in this research were all carefully selected from reliable references where considerable time has been spent on conducting experiments and calculated solubility values using specific methods, especially in three-phase conditions. Further explanation of the laboratory process for calculating gas solubility is beyond the scope of this work and interested readers are referred to the literature^6,55,56^. It should be mentioned that what is mentioned as gas solubility in this study is *x* = mole fraction of hydrocarbon gas in the aqueous liquid phase, which is collected from reliable references reported in Table 1.

**Table 1:** The solubility systems of light hydrocarbon gases in pure water and aqueous electrolyte systems.

Solubility system	Pressure (MPa)	Temperature (K)	Solubility (mole fraction)	References
Methane + pure water	0.973–17.998	275.11–313.11	C1: 0.000204–0.002459	^9^
	2–40.03	283.2–303.2	C1: 0.000563–0.004049	^24^
	2.5–100	344.25	C1: 0.000127–0.005085	^5^
	2.53–60.8	293.1–353.1	C1: 0.000361–0.004328	^25^
	4.13–34.47	310.9–344.2	C1: 0.000602–0.00335	^23^
	1.327–6.451	297.5–518.3	C1: 0.0002124–0.0010337	^20^
	9.81–113.27	423.2–633.2	C1: 0.001–0.18	^57^
	0.101325	273.15–283.15	C1: 0.0000444–0.0000345	^58^
	0.101325	273.42–353.15	C1: 0.0000188–0.0000445	^59^
Ethane + pure water	0.5–4	283.2–303.2	C2: 0.000119–0.000864	^24^
	0.8–69.61	310.92–444.26	C2: 0.0000698–0.0033	^21^
	2.5–100	344.25	C2: 0.000821–0.001398	^5^
	0.05074–0.11	275.44–323.15	C2: 0.00002073–0.0000725	^60^
	0.373–4.952	274.26–343.08	C2: 0.0000854–0.0009696	^61^
	20–370	473.15–673.15	C2: 0.005–0.34	^62^
	0.101325	285.5–345.6	C2: 0.000016– 0.0000434	^63^
Propane + pure water	0.357–3.915	277.62–368.16	C3: 0.0000321–0.0002694	^2^
	0.0995–3.409	288.7–410.9	C3: 0.0000078–0.000313	^64^
	0.49–4.269	278.87–422	C3: 0.0000796–0.000366	^65^
	0.101325	285.45–347.25	C3: 0.0000118–0.0000415	^63^
*n*-Butane + pure water	2.5–100	344.25	C4: 0.000021–0.000103	^5^
	0.12–3.044	310.9–410.9	C4: 0.000016–0.0001771	^22^
	25.5–83	628.15–637.15	C4: 0.025–0.077	^62^
	0.101325	277.15–328.15	C4: 0.000011–0.000058	^66^
Methane/ethane + pure water	1–4	275.2–283.2	C1: 0.000643–0.00115 C2: 0.000098–0.0001475	^24^
	4.58–54.572	310.9–344.2	C1: 0.00045–0.003336 C2: 0.000232–0.002439	^23^
Methane/propane + pure water	4.92–55.26	377.59	C1: 0.000862–0.003702 C3: 0.00015–0.001863	^23^
Ethane/propane + pure water	4.58–55.26	377.59	C2: 0.000208–0.000929 C3: 0.000188–0.000642	^23^
Methane/ethane/propane + pure water	4.58–34.57	344.26–377.59	C1: 0.000768–0.003276 C2: 0.000119–0.001396 C3: 0.0000019–0.000607	^23^
Methane/ethane/*n*-butane + pure water	0.987–14.407	278.14–313.12	C1: 0.000218–0.002191 C2: 0.000014–0.000067 C4: 0.00000387–0.0000112	^9^
Methane + pure water, NaCl	10.13–61.6	324.65–398.15	C1: 0.000805–0.0043	^67^
Methane + pure water, NaCl, LiCl, NaBr, NaJ, CaCl_2_	4.09–45.89	298.15–423.15	C1: 0.00017–0.00269	^68^
Methane + pure water, KCl, LiBr, KBr, LiCl	0.3–10.23	313.1–373.2	C1: 0.00003–0.00154	^4^
Methane/ethane/propane + pure water, NaCl	6.22–20.1	274.55–299	C1: 0.00099–0.0028 C2: 0.000038–0.00024 C3: 0.000006–0.000042	^6^

Literature survey reveals that the gaseous phase composition, aqueous phase composition, temperature, and pressure highly affect the solubilities of hydrocarbon gases in the aqueous solutions^1,5,6,9,68^. The ionic strength (*I*) as a single characteristic of aqueous electrolyte solutions was utilized in the modeling process instead of multiple salt concentrations of brine solutions in order to reduce the dimensions of the modeling process. Considering *m*_*i*_ as the molar concentration of each ion and *z*_*i*_ as valance of charged ions in brine solutions, the ionic strength (*I*) is defined as follows:

1 $$ {\text{I = }}\frac{1}{2}\sum {{\text{m}}_{{\text{i}}} \left| {{\text{z}}_{{\text{i}}} } \right|}^{2} $$

In this study, two approaches were considered for modeling. First, hydrocarbon gases solubility (*η*_*h*_: mole fraction) is assumed to be a function of eight independent parameters: temperature (K), pressure (MPa), ionic strength of the solution (M), the mole percent of each component (C1, C2, C3, and C4) in the gas mixture, and carbon number (*IDX*: 1, 2, 3, and 4) of the gas component (methane, ethane, propane, and *n*-butane) whose solubility is to be predicted:

2 $$ \eta_{h} = f\left( {\text{P, T, I, C1, C2, C3, C4, IDX}} \right) $$

The mentioned approach is similar to that utilized in Samani et al.^52^ and Nabipour et al.^53^ works. The second approach is that hydrocarbon gases solubility (*η*_*h*_: mole fraction) is assumed to be a function of five input parameters: pressure (MPa), temperature (K), ionic strength of the solution (M), the pseudo-critical temperature of the gas mixture (T_pc_), and the critical temperature of the gas component (Tc_gas_) whose solubility is to be predicted:

3 $$ \eta_{h} = f\left( {{\text{P, T, I, T}}_{{{\text{pc}}}} {\text{, Tc}}_{{{\text{gas}}}} } \right) $$

Here, if *Tc*_*i*_ is the critical temperature of individual components and *y*_*i*_ is the molar fraction of individual components in the gas mixture of *n* components, *T*_*pc*_ can be calculated as follows^69^:

4 $$ T_{pc} = \sum\limits_{i = 1}^{n} {y_{i} Tc_{i} } $$

In the second approach, although the number of parameters has been reduced, by using the parameters of the pseudo-critical temperature of the gas mixture and the critical temperature of gaseous components instead of the mole percent of each component in the gas mixture and the carbon number, the development of the model becomes more general. Table 2 presents the statistical details of the databank (including all inputs utilized in both modeling approaches along with hydrocarbon gases solubility as the models’ target) utilized to model the solubility of light hydrocarbon gases and their mixtures in water and aqueous electrolyte solutions.

**Table 2:** Statistical description of the solubility databank utilized in the present research.

	IDX	Temperature (K)	Pressure (MPa)	Ionic strength (M)	C1 (mole %)	C2 (mole %)	C3 (mole %)	C4 (mole %)	T_pc_ of gas mixture (K)	Tc_gas_ (k)	Solubility (mole fraction)
Mean	1.829521	341.1801	14.11	3.252	56.65336	20.70442	15.66009	6.98213	258.7715	268.3451	0.002634
SD	0.978137	64.15295	19.78	7.656	45.11362	36.41282	33.02423	24.61655	1.79276	1.96642	0.013492
Minimum	1	273.15	0.051	0	0	0	0	0	190.56	190.56	3.87E−06
Maximum	4	637.15	113.27	37.351	100	100	100	100	425.12	425.12	0.18

Table 2 reports that the ionic strength of brine solutions based on molarity is in the range of 0–37.351 M. The mole percent of light hydrocarbon gases (C1-C4) in the gaseous mixture was in the range of 0–100%. The experimental solubility data of light hydrocarbons and their mixtures in water and aqueous electrolyte systems have also been gathered over broad ranges of operating temperatures, 273.15–637.15 (K), and pressures, 0.05–113.27 (MPa). Hence, the variety of input variables is broad enough to provide a general machine learning-based predictive tool for estimating light hydrocarbon gases and their mixtures in water and aqueous electrolyte systems.

## Model development

### Adaptive boosting (AdaBoost)

The Adaptive boosting (AdaBoost) technique established by Freund and Schapire^70^ seeks to develop a powerful classifier by integrating weak classifiers and benefiting from their failures. In other words, it repeatedly chooses the training inputs in order to complement several classifiers and apply the proper weight for every classifier depending on its performance, with larger weights allocated to miscategorized data sets. The following are the common parts of the AdaBoost procedure^71^:

Step 1: Weights determination: ${w}_{j}=\frac{1}{n}. j=1.2.\dots .n$

Step 2: Providing the training data to a weak learner ${Wl}_{i}(x)$, assigning weights, and calculating the weighted error for each *i*.

$${Err}_{i}=\frac{{\sum }_{j=1}^{n}{w}_{j}I({t}_{j}\ne {wl}_{i}\left(x\right))}{{\sum }_{j=1}^{n}{w}_{j}},I\left(x\right)=\left\{\begin{array}{c}0 if x=false\\ 1 if x=true\end{array}\right.$$

Step 3: The weights should be calculated for each *i* for estimators: ${\beta }_{i}=log\left(\frac{(1-{Err}_{i})}{{Err}_{i}}\right)$

Step 4: Changing the weights of the data for each *i* to *N* (*N* refers to the count of the learner).

Step 5: Setting a weak learner to the data test (x) as a response.

Support vector regressors are utilized as weak learners in the AdaBoost algorithm in this research.

### Support vector machine for regression (SVR)

Although support vector machine is a collection of controlled machine learning techniques that may be applied for regression and classification^72^, support vector regression (SVR) is routinely used for soft calculation since it has a well-defined mathematical model. Because of its consistency in simulating numerous complicated structures, SVR has recently piqued researchers’ curiosity. Since the main theory of SVR has been published^73^, it is just shortly presented in this work for the sake of brevity. The SVR objective is to catch a regressor *f(x)* for such a sample data $[\left({x}_{1}. {y}_{1}\right).\dots ..\left({x}_{n}.{y}_{n}\right)]$, having $x\in {R}_{d}$ as the d-dimensional input dataset and $y\in R$ as the output variable (which relies on the inputs), in order to calculate the output:

5 $$f\left(x\right)=w.\phi \left({x}_{i}\right)+b$$

Here *w* denotes weight, *b* indicates bias vectors, and $\phi \left(x\right)$ represents the kernel function. To get the proper aforementioned parameters, Vapnik et al.^74^ developed the following minimizing method:

$$minimize \frac{1}{2}{w}^{T}w+C\sum_{j=1}^{N}\left({\zeta }_{j}^{-}+{\zeta }_{j}^{+}\right)$$

6 $$\left\{\begin{array}{l}\left(w.\varnothing \left({x}_{i}\right)+b\right)-{y}_{i}\le \varepsilon +{\zeta }_{j}^{-}\\ {y}_{i}-\left(w.\varnothing \left({x}_{i}\right)+b\right)\le \varepsilon +{\zeta }_{j}^{+}\\ {\zeta }_{j}^{+}.{\zeta }_{j}^{-}\ge 0 . i=1.2\dots .m\end{array}\right.$$

where transposed matrix of *w* is represented by ${w}^{T}$, error connivance by $\varepsilon$, positive factors expressing the lower and higher extra variances by ${\upzeta }_{\mathrm{j}}^{+}$ and ${\upzeta }_{\mathrm{j}}^{-}$, and positive regularization parameter indicating the variation from $\varepsilon$ by *C*.

**Figure 1:**
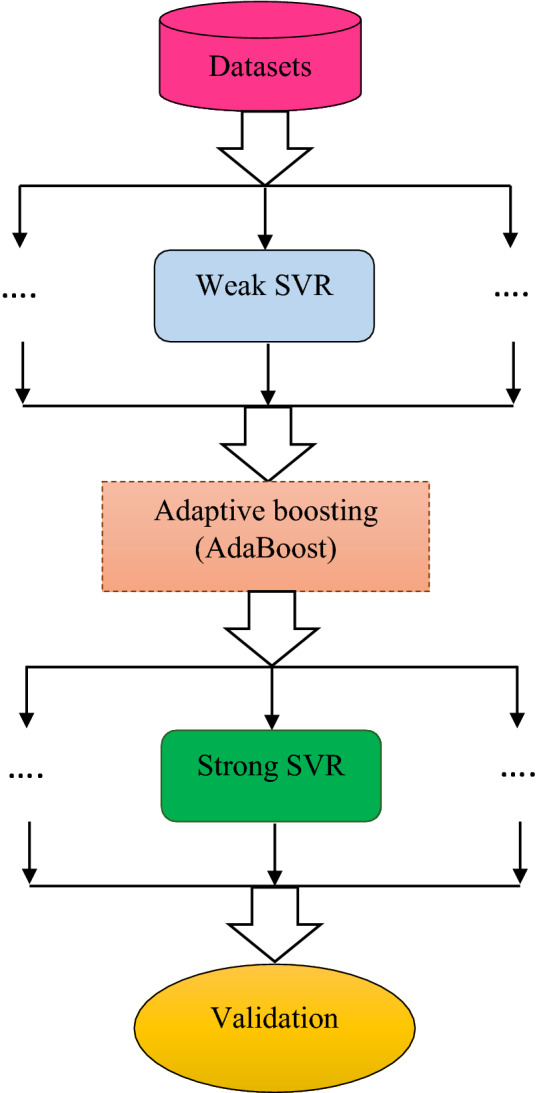
Schematic illustration of the proposed AdaBoost-SVR.

The abovementioned constraints optimization issue is transformed into a dual function utilizing Lagrange multipliers, yielding the subsequent solution:

7 $$ f(x) = \sum\limits_{j = 1}^{n} {(a_{k} - a_{k}^{*} )K(x_{k} ,x_{l} ) + } b $$

where ${a}_{k}^{*}$ and ${a}_{k}$ indicate the Lagrange multipliers, while $K\left({x}_{k}.{x}_{l}\right)$ is the kernel function. Figure 1 presents a schematic image of the proposed AdaBoost-SVR in this study.

### Decision tree (DT)

**Figure 2:**
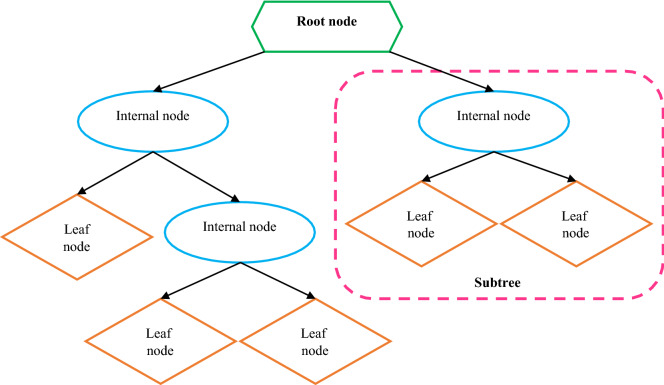
Schematic illustration of a typical decision tree.

This method^75^ is derived from natural sources and may be used to tackle both regression and classification problems. Root nodes, leaf nodes, internal nodes, and branches make up this system. The inputs are carried by the root node, which is the initial portion of the proposed technique. The last section of the diagram, known as the leaf nodes or final nodes, represents the model's output. Between the root and leaf nodes are internal nodes. The nodes are linked together by branches. Pruning, dividing, and halting are the three major activities used to build a decision tree^76^. The data dividing stage begins from the root node just before data is presented to the system. This process of separating proceeds until a stopping condition is met^77^. Figure 2 depicts the basic DT.

### Random forest (RF)

**Figure 3:**
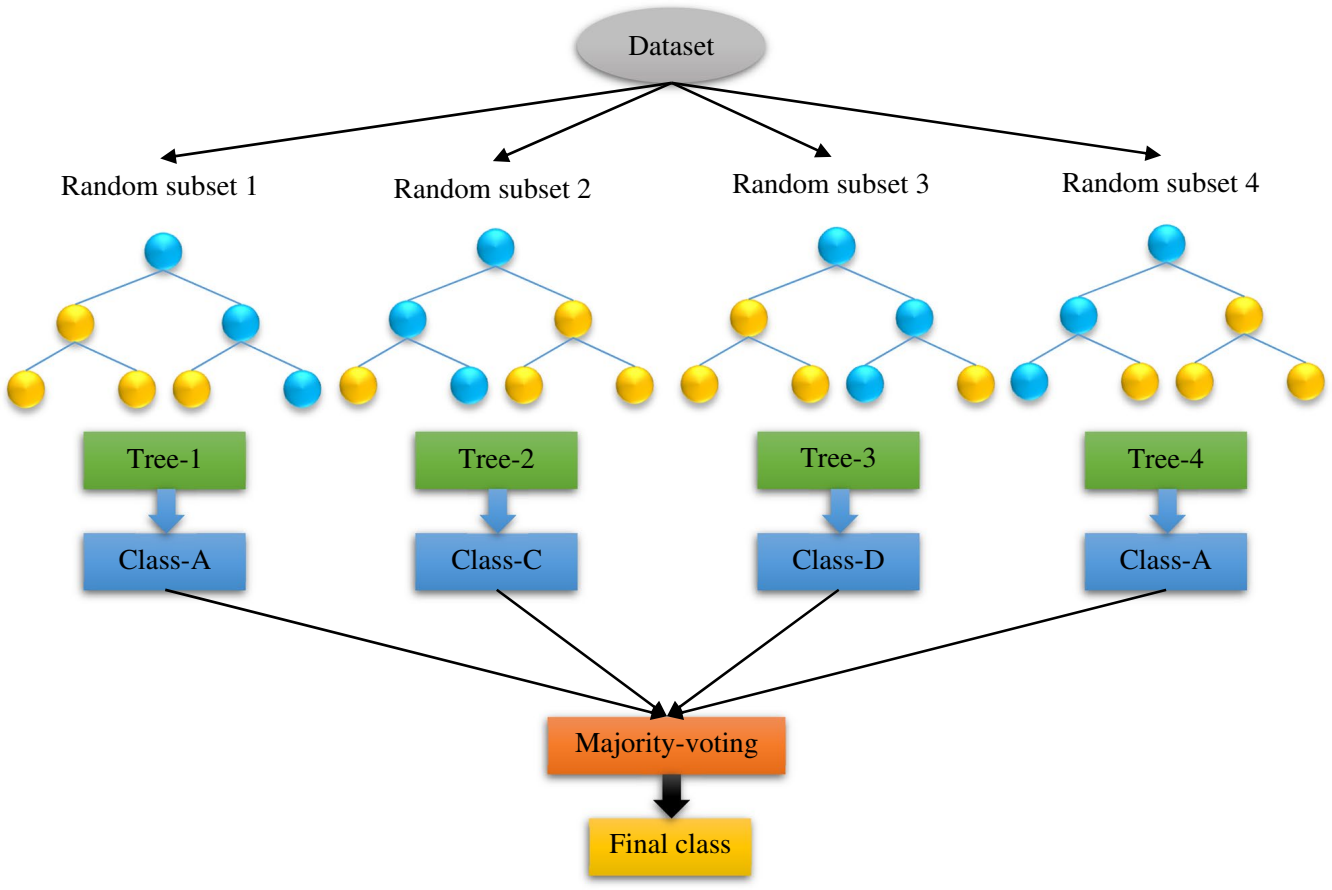
A schematic of the random forest model.

The decision tree is an effective machine learning technique; however, it has two flaws. First, while the estimation error of the decision tree is typically low in training data, the forecasting deviation is sometimes high because it is susceptible to small disturbances in the training samples; second, while the separating law in each node is desirable, according to the previous section, this greedy strategy cannot assure that the overall decision tree is the best. By simultaneously training many trees and transforming several weak learners into powerful learners, ensemble techniques can address these two problems. A random forest is made up of a set of different decision trees that are all being learned at the same time. The system determines the superiority and significance of each decision tree^78^. Furthermore, a constructed attribute of the Classification model that is used to choose different attributes allows the RF to govern various inputs characteristics without the requirement to remove a set of variables for dimension decrement ^79^. The RF approach uses a process called Bagging throughout the simulation to increase the variety of trees in the forest. Usually, the system provides the number of trees as an input, and the algorithm divides datasets into distinct groupings as a result. Bagging is a sort of sample selection approach that uses only a third of the datasets in the learning phase of the subtree creation procedure, with the other inputs being known as the out-of-bag data (OOB). Moreover, verification of outputs is not necessary for the RF during model building since the correctness of the model may be assessed utilizing OOB's errors^80^. The RF technique is shown in Fig. 3. If the system is provided with a training dataset as a prerequisite, the training procedure will be completed. If you have a training sample in the form of $D=[\left({x}_{1}.{y}_{1}\right).\left({x}_{2}.{y}_{2}\right).\ldots \left({x}_{n}.{y}_{n}\right)]$, ${D}_{t}$ is the described training data for tree ${h}_{t}$, and the final estimation of the out-of-bag dataset of sample *x* is ${H}^{oob}$, as shown:

8 $${H}^{oob}\left(x\right)=argmax{\sum }_{t=1}^{T}I({h}_{t}\left(x\right))=y$$

The error of the OOB data is extended as following for modeling purposes:

9 $${\varepsilon }^{oob}\left(x\right)=\frac{1}{\left|D\right|}{\sum }_{(x.y)\epsilon D}I({H}^{oob}(x)\ne y)$$

The functioning of the RF must be randomized, and this characteristic is regulated by the variable $k={log}_{2}d$^80^. The following equation may be used to determine the importance of a feature of a parameter *X*_*i*_:

10 $$I\left({X}_{i}\right)=\frac{1}{B}{\sum }_{t}^{B}\widetilde{OOBe}r{r}_{{t}^{i}}-OOBer{r}_{t}$$

Correspondingly, the *i*th component is characterized by *X*_*i*_ in the *X* vector, *B* represents the number of trees in the existing RF, the original OOB datasets are offered as the $OOBer{r}_{t}$, which involves the replaced parameters, and the estimated error of the OOB samples is described by $\widetilde{OOB}er{r}_{{t}^{i}}$, which refers to the attribute *X*_*i*_ of tree *t*.

### Extra tree (ET)

The Extra trees ^81^ are a novel machine learning approach that was created as an improvement of the random forest model and is less prone to over-fit a database^81^. Extra tree (ET) randomly selects a set of attributes to train a basic predictor^82^, using the same idea as random forest. For dividing the node, it chooses the best characteristic and the matching value at random^82^. For every regression tree, ET utilizes all training data. In contrast, RF's model is trained using a bootstrap replica.

### Genetic programming (GP)

GP is an organized method for getting machines to automatically solve a problem beginning with a high-level statement of what ought to be accomplished. GP is a systematic approach that is independent of a problem domain, that genetically reproduces a population of programs to solve a problem^83,84^. Programs are ‘bred’ through the continuous progress of an initially random population of programs. Actually, in this iterative improvement approach, at each new step of the algorithm, it selects only the fittest of the descendant to pass and regenerate in the subsequent production, which is occasionally referred to as a fitness function^85^. More explanations related to the application of this algorithm in the implementation of symbolic regression can be found elsewhere in the literature^86–88^.

### Group method of data handling (GMDH)

GMDH^89^ features fully automatic structural and parametric optimization of models and is a kind of inductive algorithm for computer-based mathematical modeling of multi-parametric datasets. In the inner levels of the GMDH method^90^, there are multiple independent neurons. All neurons per layer are attached in couples via a quadratic polynomial and form individual neurons in the structure of polynomials in the subsequent layer^91^. Each GMDH neuron's generated value is determined by employing a quadratic polynomial representative that comprises the preceding neuron^92,93^. The quadratic polynomial procedures merging the neurons in the earlier levels will create the neurons in the subsequent layers^94^. To amend the limitations of the primary GMDH method^89^, the hybrid GMDH is usually utilized which has more than two independent variables that can be combined concurrently and it permits the intersection of nodal within diverse layers. The succeeding formula shows the final form of the hybrid GMDH^95^:

11 $$ {\text{Y}}_{{\text{i}}} = {\text{a }} + \sum\limits_{i = 1}^{M} {\sum\limits_{j = 1}^{M}\cdots  { \sum\limits_{k = 1}^{M} {b_{ij \ldots k} } } } x_{i}^{n} x_{j}^{n}  \ldots x_{k}^{n}  \quad n = 1,2,\ldots,2^{l} $$

Here, *M* is the count of inputs, *l* stands for the count of layers, *x*_*i*_*, x*_*j*_*, …, x*_*k*_ are the inputs, *a, b*_*ij…k*_ denote the polynomial coefficients, and *Y* indicates the model output.

### Equations of state (EOSs)

An EOS is utilized to relate pressure, volume, and temperature (PVT) for both systems of a pure substance and for multi-component mixtures. There are many EOSs in the thermodynamic literature that is used to describe vapor–liquid-equilibria, solubility estimation, thermal features, and volumetric properties of a substance or multi-component mixtures^71^. In this work, three famous EOSs, namely SRK, VPT, and PR, have been utilized to estimate the solubility of light hydrocarbon gases in water with the purpose of comparing them with machine learning algorithms. Tables S1 in the Supplementary file presents the PVT relationships of these EOSs. Also, the parameters of considered EOSs are presented in Table S2. Besides, acentric factors and critical properties of the light hydrocarbon gases and water are represented in Table S3 used in EOSs.

## Assessment of models

The following statistical factors viz., determination coefficient (R^2^), average absolute percent relative error (AAPRE), root mean square error (RMSE), and standard deviation (SD) were employed to assess the accuracy of the machine learning models. The mathematical formula of these statistical criteria is defined below^96,97^:

12 $$ RMSE = \sqrt {\frac{1}{N}\sum\limits_{i = 1}^{N} {\left( {\eta_{i,\exp } - \eta_{i,pred} } \right)}^{2} } $$

13 $$ R^{2} = 1 - \frac{{\sum\limits_{i = 1}^{N} {(\eta_{i,\exp } - \eta_{i,pred} )^{2} } }}{{\sum\limits_{i = 1}^{N} {(\eta_{i,\exp } - \overline{{\eta_{\exp } }} )^{2} } }} $$

14 $$ AAPRE = \frac{100}{N}\sum\limits_{i = 1}^{N} {\left| {\frac{{\eta_{i,\exp } - \eta_{i,pred} }}{{\eta_{i,\exp } }}} \right|} $$

15 $$ SD = \sqrt {\frac{1}{N - 1}\sum\limits_{i = 1}^{N} {\left( {\frac{{\eta_{i,\exp } - \eta_{i,pred} }}{{\eta_{i,\exp } }}} \right)}^{2} } $$

where *N* refers to the count of data, *η*_*i,exp*_ shows the experimental hydrocarbon gases solubility, and *η*_*i,pred*_ is predicted hydrocarbon gases solubility in the liquid phase by presented models.

In the present research, the subsequent graphical analyses are utilized simultaneously to assess the performance of machine learning-based models and correlations:

Histogram plot: in this graph, the discrepancy between the experiments data and prediction of the model can be seen statistically, which helps to evaluate the model's performance.

Cross-plot: the cross-plot graph illustrates the correlation between experimental solubilities and predicted values by models with the fact that the higher the concentration of data nearby the unit-slope line, the better the model's prediction.

Error distribution plot: the scatter of error (exp-pred) around the zero-error line is evaluated to check for possible error trends.

Trend plot: the experiments data and prediction of the model are plotted versus a special property to assess the model's validation by checking the coverage of these data. High data coverage shows the high validity of the model.

Cumulative frequency graph: it is a statistical plot for quantifying the precision of the models, which is shown by drawing the cumulative frequency of data against absolute error (exp-pred).

## Results and discussion

### Correlations’ development

As mentioned earlier, this work employed white-box modeling approaches to create precise predictive correlations for the solubility of light hydrocarbon gases and their mixture in brine. The correlations utilize the second modeling approach having five inputs (P, T, I, T_pc_ of gas mixture, Tc_gas_) to calculate hydrocarbon gases solubility. The reason for choosing five parameters for the development of mathematical correlations was that, firstly, a simpler mathematical expression was obtained and solubility calculations become easier, and secondly, the correlation become more general by using the pseudo-critical of the gas mixture instead of using the percentage of gas (C1–C4) composition. The proposed correlations by GMDH and GP methods are presented below:

GMDH correlation:

$$Solubility = -0.000257478 + {N}_{6}*0.104357 + {N}_{1}*0.995504$$

$${N}_{1}= -0.000402032 + P*3.34159e-05 + {N}_{2}*0.976721$$

$${N}_{2} = 0.000417773 + {N}_{5}*0.163256 + {N}_{3}*0.277835 + {{N}_{3}}^{ 2} *6.25097$$

$${N}_{3} = 0.000769644 + {N}_{4}*{N}_{5}*81.1485 - {{N}_{4}}^{2} *31.6265 - {{N}_{5}}^{ 2} *30.9349$$

$${N}_{4} = 0.0113595 - {T}^{2}*1.51522e-07 + {\text{T}}*P*3.24299e-09 + {\text{T }}^{4}*4.06799e-13 - P*0.000290132 - {\text{P}}^{ 2}*1.23427e-06$$

16 $$N5 = 0.00995312 + {\text{Tc}}{,}^{2}*4.48223e-08 - {\text{Tc}}^{2}*{T}_{pc}^{2} \, *5.36312e-13 + {\left({\text{Tc}}\right)}^{4}*3.23202e-14 - {T}_{pc}^{2}*1.85458e-07 + {T}_{pc}^{4}*9.26622e-13$$

$$N6= 0.0128381 - {\text{Tc}}^{2} *2.05784e-07 + \, {\text{Tc}}^{2}*I*5.76622e-09 + {\left({\text{Tc}}\right)}^{4}*8.16174e-13 - I*0.00081115 + {\text{I}}^{ 2}*1.35367e-05$$

GP correlation:

17 $$Solubility= \left(\left(\frac{\mathrm{log}(\mathrm{log}\left({c}_{0}P+{c}_{1}\right))}{\frac{{c}_{2}Tc}{\mathrm{exp}(\frac{{(c}_{3}T)}{{c}_{4}I})}}-(\mathrm{exp}\left({c}_{5}\right)\mathrm{exp}\left({c}_{6}T\right)-\left({c}_{7}{T}_{pc}+\mathrm{log}\left(\mathrm{log}\left(\mathrm{log}\left(\left({c}_{8}P+{c}_{9}\right)\right)\right)\right)\right)\right){c}_{10}+{c}_{11}\right)$$

$${c}_{0}=0.909;{c}_{1}=-19.076;{c}_{2}=0.45799;{c}_{3}=0.6495;{c}_{4}=15.867;{c}_{5}=4.777;{c}_{6}=0.026667;{c}_{7}=0.87809;{c}_{8}=0.909 ;{c}_{9}=-19.194;{c}_{10}=9.7169E-12;{c}_{11}=0.0018755$$

### Evaluation of the models

In the current study, R^2^, AAPRE, SD, and RMSE were utilized to appraise the models' estimates. The results of these statistical criteria for all predictive tools are presented in Table 3. As can be observed in this table, for both modeling approaches, AdaBoost-SVR, Extra Tree, Random Forest, and DT models can be classified in terms of high exactness for predicting the whole dataset, respectively. However, for the test subset, AdaBoost-SVR, Random Forest, DT, and Extra Tree models, respectively, had the best estimates, which is the most important part of the assessment of models. AAPRE values of 10.64% for the total collection, 11.49% for the test collection, and 10.43% for the train collection, as well as a total R^2^ value of 0.9999, indicating that the AdaBoost-SVR model developed with 8 inputs had the most precise predictions of hydrocarbon gases solubilities in aqueous electrolyte solutions. After that, in terms of accuracy, the AdaBoost-SVR model developed with 5 inputs with an AAPRE of 12.02% for the total collection and a total R^2^ value of 0.9999 ranks second among all models. AdaBoost-SVR models have the least overall values of RMSE, SD, and AAPRE along with the highest overall R^2^ value among the other machine learning models leading us to conclude that this model is the most accurate model for predicting light hydrocarbon gases and their mixtures in water and aqueous electrolyte solutions. Moreover, despite the expected poorer performance than machine learning models, the mathematical correlations yielded by GP and GMDH methods show satisfying results with AAPRE values of 16.44% and 20.95%, respectively.

**Table 3:** Statistical error analysis for the developed models and correlations.

Statistical criteria		RMSE	SD	R^2^	AARPE, %
Random forest (8 inputs)	Train	0.001099	0.47198	0.9928	15.092
	Test	0.001628	0.47280	0.9886	16.089
	Total	0.001223	0.47217	0.9917	15.292
Decision tree (8 inputs)	Train	0.000154	0.27784	0.9998	17.019
	Test	0.000383	0.63358	0.9991	20.762
	Total	0.000220	0.37761	0.9997	17.769
AdaBoost-SVR (8 inputs)	Train	0.000099	0.20911	0.9999	10.433
	Test	0.000101	0.25008	0.9999	11.497
	Total	0.000099	0.21807	0.9999	10.647
Extra tree (8 inputs)	Train	0.000218	0.23459	0.9997	11.979
	Test	0.002642	0.69527	0.9585	25.802
	Total	0.001199	0.37821	0.9921	14.750
Random forest (5 inputs)	Train	0.001099	0.61834	0.9928	15.365
	Test	0.001803	0.37921	0.9860	14.314
	Total	0.001272	0.57841	0.9911	15.154
Decision tree (5 inputs)	Train	0.000170	0.43871	0.9998	18.313
	Test	0.000391	0.85103	0.9991	21.875
	Total	0.000231	0.54727	0.9997	19.027
AdaBoost-SVR (5 inputs)	Train	0.000102	0.25916	0.9999	11.613
	Test	0.000109	0.44120	0.9999	13.643
	Total	0.000104	0.30470	0.9999	12.020
Extra tree (8 inputs)	Train	0.000331	0.22614	0.9994	11.413
	Test	0.002457	1.06098	0.9642	31.982
	Total	0.001138	0.52128	0.9928	15.536
GMDH correlation (5 inputs)	Train	0.001973	1.06744	0.9769	17.470
	Test	0.006485	0.88234	0.8190	34.834
	Total	0.003397	1.03387	0.9365	20.951
GP correlation (5 inputs)	Train	0.002456	0.57392	0.9643	13.640
	Test	0.006386	0.53905	0.8245	27.615
	Total	0.003605	0.56727	0.9286	16.441

In the next step, the performance of the machine learning algorithms was compared with SRK, PR, and VPT EOSs. To this end, the solubilities data of light hydrocarbon gases in pure water at different operating conditions, acquired from the literature^2,9,22,61^, was predicted by the developed machine-learning models, mathematical correlations, and three EOSs. Table 4 reports the predictions of these predictive tools and EOSs as well as calculated AAPRE. Aa represented in Table 4, AdaBoost-SVR models are superior to all machine learning-based predictive tools and EOSs showing AAPRE values of 5.13% (AdaBoost-SVR model with 5 inputs) and 5.45% (AdaBoost-SVR model with 8 inputs), which is the least among these predictive tools. Among the EOSs, VPT, SRK, and PR are ranked in terms of good predictions, respectively. Moreover, the mathematical correlations generated by the GMDH and GP techniques demonstrate satisfactory results with an AAPRE of approximately 10%.

**Table 4:** Estimates of EOSs, mathematical correlations, and machine-learning models for the solubilities of light hydrocarbon gases in pure water.

Solubility system	Data No.	P (MPa)	Gas solubility, mole fraction													
			Exp	DT (8 inputs)	Extra tree (8 inputs)	AdaBoost-SVR (8 inputs)	Random forest (8 inputs)	DT (5 inputs)	Extra tree (5 inputs)	AdaBoost-SVR (5 inputs)	Random forest (5 inputs)	GMDH correlation (5 inputs)	GP correlation (5 inputs)	PR	SRK	VPT
Methane + water, at 275 K^9^	1	0.973	0.000399	0.000263	0.000349	0.000393	0.000324	0.000263	0.000351	0.000379	0.000363	0.000331	0.000364	0.000298	0.000302	0.000351
	2	1.565	0.000631	0.000668	0.000524	0.000666	0.000784	0.000667	0.000581	0.000666	0.000737	0.000559	0.000593	0.000401	0.000703	0.000553
	3	2.323	0.000901	0.000668	0.000636	0.000863	0.000901	0.000668	0.000694	0.000866	0.000787	0.000764	0.000966	0.000608	0.001005	0.000805
	4	2.82	0.001061	0.000668	0.000624	0.000939	0.000919	0.000669	0.000698	0.000944	0.000902	0.000766	0.001046	0.000802	0.001204	0.000947
Ethane + water, at 303 K^61^	5	0.373	0.000134	0.000192	0.000150	0.000138	0.000170	0.000192	0.000156	0.000141	0.000134	0.000157	0.000171	0.000103	0.000102	0.000101
	6	0.719	0.000240	0.000192	0.000225	0.000245	0.000210	0.000192	0.000247	0.000246	0.000197	0.000240	0.000222	0.000193	0.000201	0.000205
	7	1.093	0.000346	0.000412	0.000353	0.000346	0.000388	0.000415	0.000356	0.000347	0.000355	0.000328	0.000275	0.000284	0.000296	0.000311
	8	1.598	0.000472	0.000675	0.000492	0.000491	0.000511	0.000675	0.000462	0.000487	0.000522	0.000452	0.000471	0.000396	0.000414	0.000414
	9	2.299	0.000630	0.000675	0.000598	0.000611	0.000616	0.000676	0.000570	0.000620	0.000606	0.000623	0.000629	0.000487	0.000508	0.000539
	10	2.932	0.000742	0.000675	0.000694	0.000734	0.000728	0.000677	0.000654	0.000740	0.000722	0.000727	0.000741	0.000584	0.000610	0.000638
	11	3.977	0.000883	0.000685	0.000755	0.000844	0.000800	0.000679	0.000731	0.000844	0.000767	0.000812	0.000882	0.000680	0.000702	0.000802
Propane + water, at 368 K^2^	12	0.41	0.000032	0.000053	0.000047	0.000046	0.000052	0.000053	0.000047	0.000042	0.000045	0.000041	0.000060	0.000027	0.000028	0.000027
	13	1.028	0.000089	0.000114	0.000105	0.000096	0.000110	0.000114	0.000105	0.000093	0.000114	0.000095	0.000089	0.000073	0.000075	0.000073
	14	1.433	0.000120	0.000114	0.000134	0.000122	0.000135	0.000115	0.000131	0.000121	0.000137	0.000123	0.000121	0.000100	0.000102	0.000102
	15	1.94	0.000159	0.000150	0.000169	0.000167	0.000177	0.000150	0.000170	0.000168	0.000173	0.000161	0.000158	0.000129	0.000132	0.000139
	16	2.495	0.000199	0.000202	0.000202	0.000205	0.000204	0.000202	0.000209	0.000203	0.000204	0.000205	0.000181	0.000156	0.000160	0.000170
	17	2.997	0.000224	0.000259	0.000226	0.000228	0.000232	0.000258	0.000230	0.000225	0.000235	0.000230	0.000230	0.000176	0.000181	0.000193
	18	3.503	0.000248	0.000271	0.000249	0.000249	0.000257	0.000233	0.000246	0.000288	0.000263	0.000311	0.000254	0.000214	0.000210	0.000212
	19	3.915	0.000260	0.000271	0.000253	0.000257	0.000259	0.000234	0.000256	0.000257	0.000274	0.000261	0.000266	0.000230	0.000223	0.000221
*n*-Butane + water, at 410 K^22^	20	0.2792	0.000022	0.000013	0.000052	0.000027	0.000020	0.000013	0.000041	0.000025	0.000019	0.000033	0.000018	0.000033	0.000032	0.000027
	21	1.003	0.000076	0.000114	0.000087	0.000074	0.000093	0.000114	0.000082	0.000073	0.000081	0.000072	0.000063	0.000059	0.000058	0.000056
	22	1.486	0.000110	0.000114	0.000117	0.000107	0.000110	0.000114	0.000112	0.000106	0.000116	0.000105	0.000086	0.000096	0.000093	0.000088
	23	1.727	0.000123	0.000150	0.000124	0.000122	0.000121	0.000151	0.000124	0.000124	0.000127	0.000120	0.000116	0.000111	0.000109	0.000103
	24	2.43	0.000157	0.000163	0.000156	0.000157	0.000163	0.000155	0.000158	0.000158	0.000159	0.000151	0.000161	0.000150	0.000142	0.000133
	25	3.044	0.000177	0.000163	0.000171	0.000173	0.000177	0.000177	0.000175	0.000176	0.000177	0.000166	0.000177	0.000173	0.000164	0.000158
AAPRE, %				21.20	16.04	5.45	11.46	20.91	13.19	5.13	9.79	10.06	10.02	20.05	17.07	15.02

**Figure 4:**
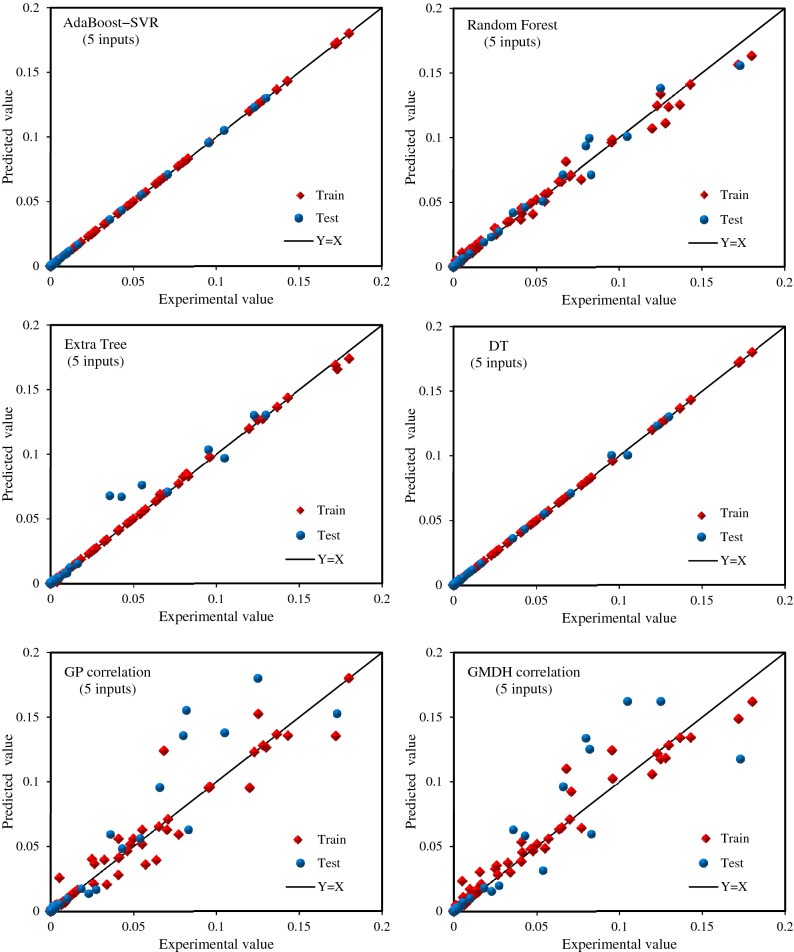

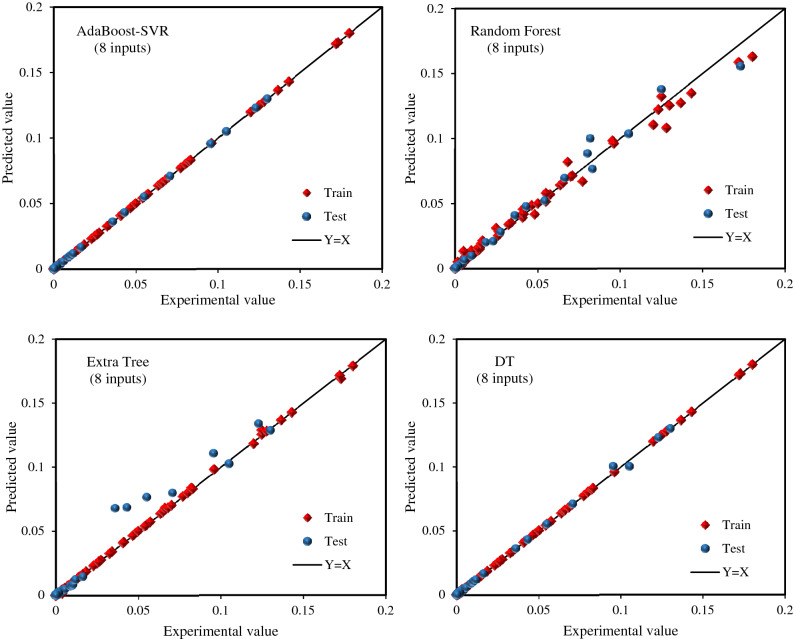
Cross-plots of the developed machine learning models and mathematical correlations.

To gain a better vision of the validity of the machine learning models in the training and testing stages, graphical error analyses were conducted along with statistical analyses. First, cross plots of all models are compared in Fig. 4. As pointed out earlier, the nearer the data to the X = Y line, the greater precision of the model in prognosticating hydrocarbon gases and their mixtures in water and aqueous electrolyte systems. As can be observed in Fig. 4, the AdaBoost-SVR models (developed with 8 and 5 inputs) have the high closest data around the X = Y line compared to the other suggested models and correlations, which exhibits the great robustness and validness of these models for the prediction of hydrocarbon gases solubility in aqueous electrolyte systems. However, other models have also performed well. Next, the error distribution graphs of all developed predictive tools based on temperature and pressure are illustrated in Fig. S1 in the supplementary file. These plots help to distinguish the performance of the models at different pressures and temperatures. Fig. S1(a) shows the low scatter of errors around the zero-error line for all models at different pressures, especially AdaBoost-SVR and DT models. Fig. S1(b) demonstrates that the AdaBoost-SVR models have the least scattering of errors around the zero-error line compared to other models and correlations at different temperatures. In relation to Random Forest, Extra Tree, and GMDH models, it seems that although the predictions of these models show a low error at low temperatures, at high temperatures, the scattering of error is high. Overall, the AdaBoost-SVR models are superior to other machine learning models in different temperature and pressure ranges.

**Figure 5:**
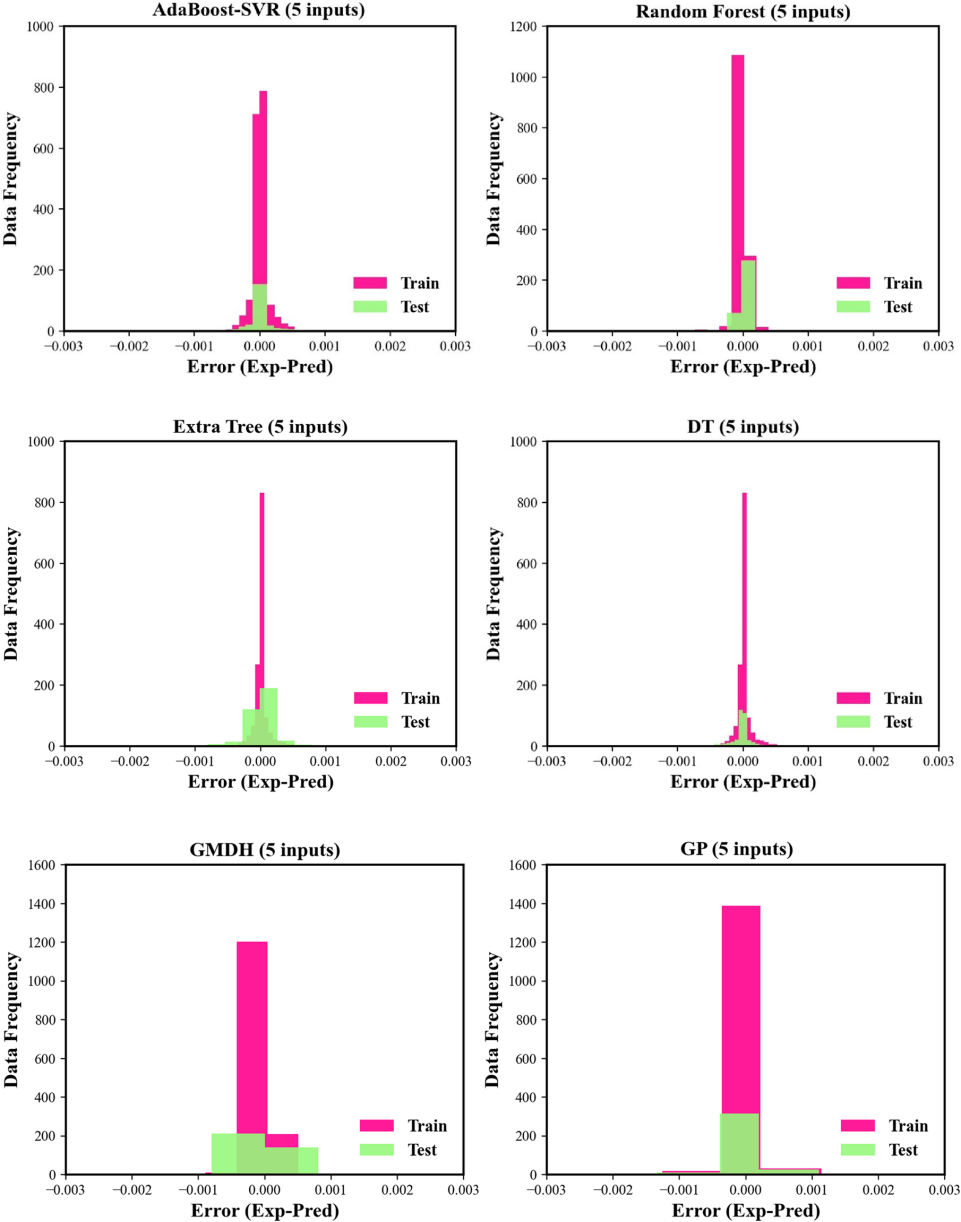

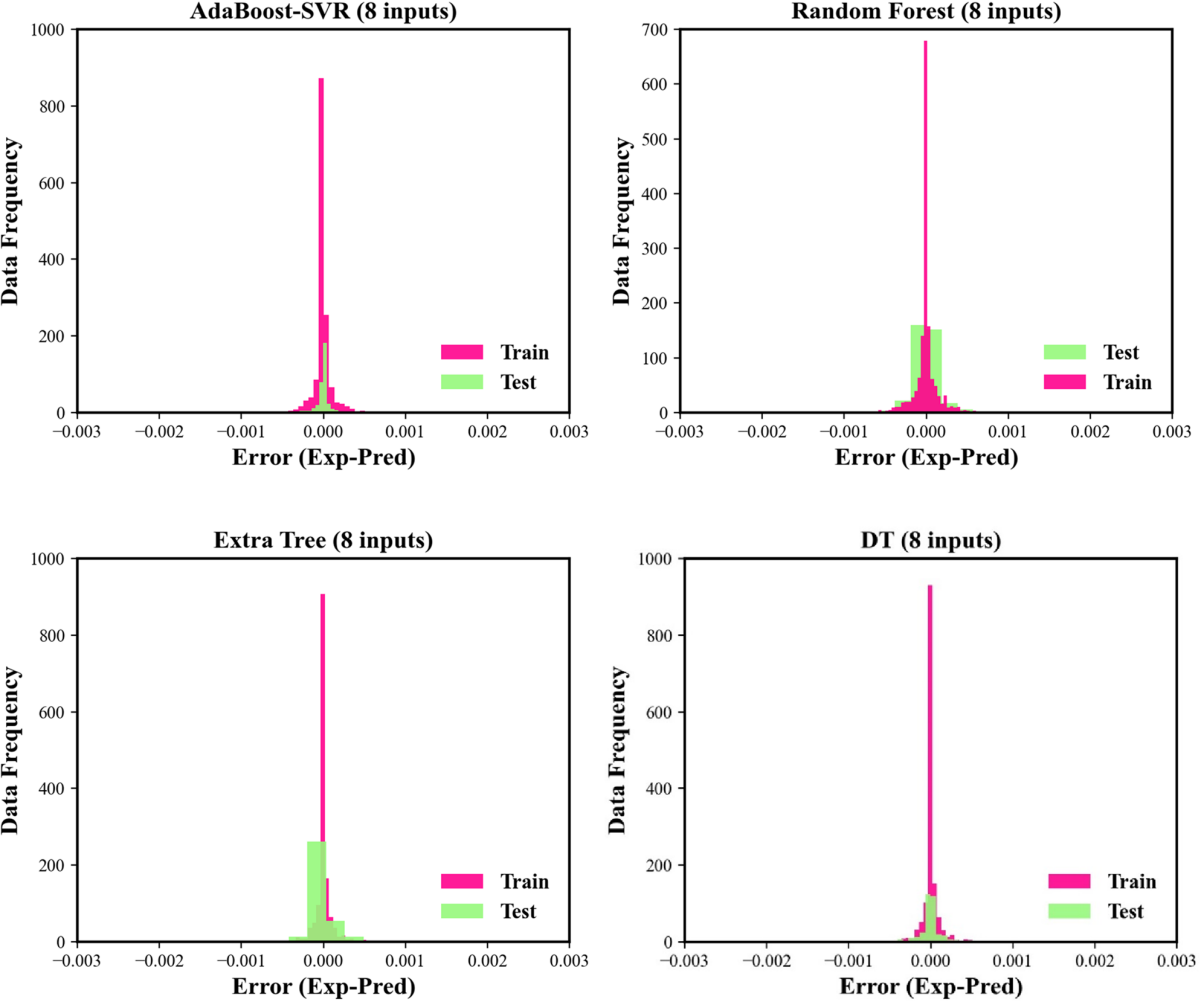
Histograms of residuals for the machine learning models and correlations.

In the next step, the histograms of errors between experimental solubilities and prognosticated values associated with all models are illustrated in Fig. 5. The computed error values for all models are located in a narrow scope from −0.001 to 0.001. This figure shows that the histograms of all machine learning models benefit from normal distributions. However, despite the excellent performance in the training phase, the histogram of the Extra Tree model seems to be a bit skewed in the testing phase. As can be observed in Fig. 5, all histogram plots benefit from the bursts of growing at zero-error value, which indicates the excellent match between the estimated solubility data and experimental values. However, again AdaBoost-SVR and DT models display less error for more data during both testing and training stages in both modeling approaches.

**Figure 6:**
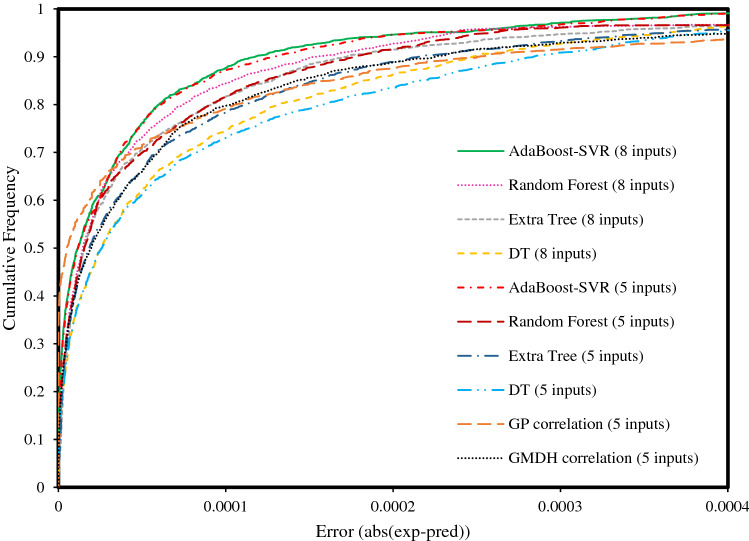
Cumulative frequency plot of the proposed predictive tools for estimating the solubility of hydrocarbon gases.

The next step of graphical error analysis is a helpful statistical plot for quantifying the precision of the models and correlations, named cumulative frequency plot. As shown in Fig. 6, the cumulative frequency curves of the AdaBoost-SVR models are very close to the vertical axis, which indicates the high accuracy of these models. Besides, more than 70% of predicted gas solubility data by the AdaBoost-SVR models have an absolute error of less than 0.00004, and more than 90% of the predicted data have an error of less than 0.00013. Meanwhile, other models and correlations including Extra Tree, DT, Random Forest, GP, and GMDH represent absolute errors of 0.00015–0.0003 for 90% of the data, respectively. Therefore, this conclusion can be drawn that the AdaBoost-SVR models are superior to other models and correlations in estimating the solubility of hydrocarbon gases and their mixtures in water and aqueous electrolytes.

According to the results of statistical and graphical analyses of machine learning models, it can be concluded that the AdaBoost-SVR models (developed with 8 and 5 inputs) are more precise in estimating the solubility of hydrocarbons in water and brine solutions than other models suggested in this work. To assess the accuracy of the proposed AdaBoost-SVR models against the available predictive models in the literature for estimating the solubility of hydrocarbon gases, the AdaBoost-SVR results were compared with two machine learning models, including Samani et al.^52^ and Nabipour et al.^53^, which are shown in Table 5. As depicted in Table 5, the AdaBoost-SVR models proposed in this study have the lowest AAPRE values plus the highest R^2^ value, indicating that the AdaBoost-SVR models are more precise than other artificial intelligence models presented in the literature for estimating the solubility of hydrocarbon gases.

**Table 5:** Statistical factors for the available hydrocarbon gases solubility predictive models and the proposed AdaBoost-SVR models.

Models		RMSE	R^2^	AARPE, %
Samani et al.^52^	Train	0.00013	0.9893	28.78
	Test	0.00017	0.9834	37.84
	Total	0.00014	0.9880	30.60
Nabipour et al.^53^	Train	0.0001	0.9850	22.049
	Test	0.0001	0.9870	22.054
	Total	0.0001	0.9850	22.050
AdaBoost-SVR (8 inputs)	Train	0.000099	0.9999	10.433
	Test	0.000101	0.9999	11.497
	Total	0.000099	0.9999	10.647
AdaBoost-SVR (5 inputs)	Train	0.000102	0.9999	11.613
	Test	0.000109	0.9999	13.643
	Total	0.000104	0.9999	12.020

### Trend analysis

**Figure 7:**
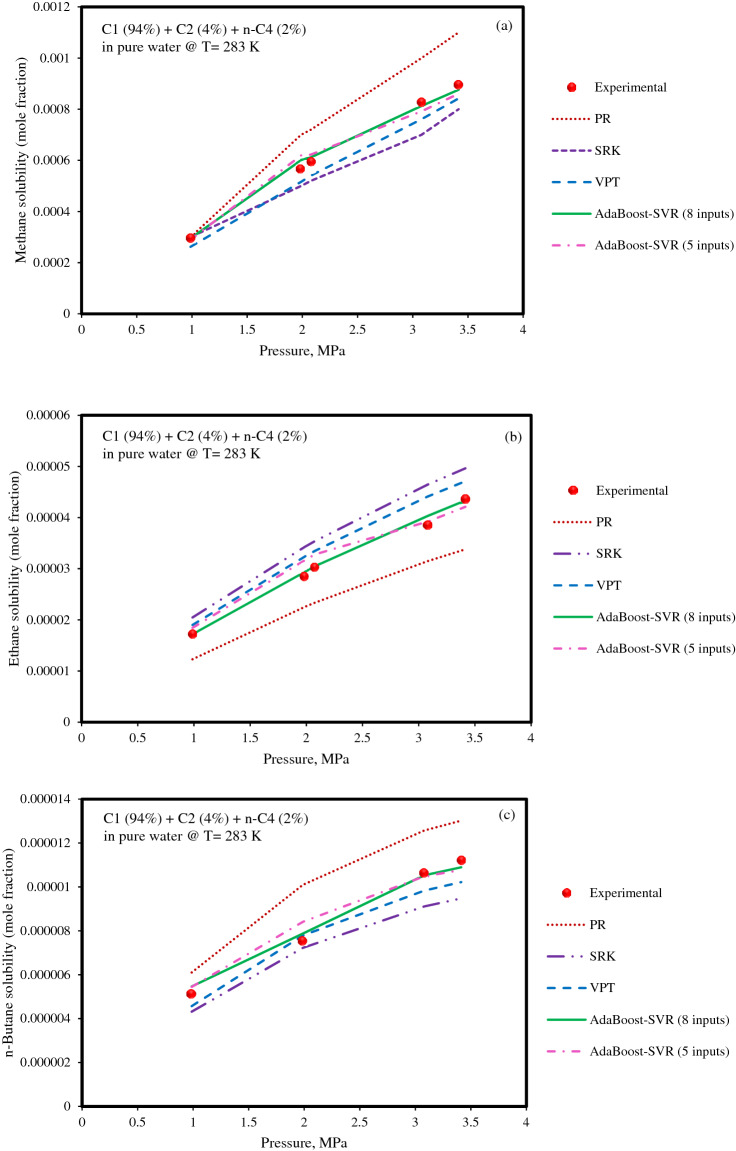
Experimental values and estimations of the solubilities of (**a**) methane, (**b**) ethane, and (**c**) *n*-butane in the aqueous phase of the gas mixture + water system by EOSs and AdaBoost-SVR models.

As mentioned earlier, the AdaBoost-SVR models are more accurate in predicting the solubility of light hydrocarbon gases in aqueous solutions than other models. Hence, the solubilities of hydrocarbon gases in several solubility systems have been investigated to evaluate the ability of the AdaBoost-SVR models in estimating the true physical trend of gases solubility in the liquid phase. In the beginning, the solubilities of methane, ethane, and *n*-butane in a gas mixture + pure water system at a temperature of 283 K^9^ were estimated utilizing the AdaBoost-SVR models and three EOSs, and the outcomes are depicted in Fig. 7. As demonstrated in Fig. 7, EOSs overestimated or underestimated the solubilities of hydrocarbon gases in water at low-temperature conditions. However, VPT EOS again is superior to SRK and PR EOSs and provides better estimations. Nevertheless, both AdaBoost-SVR models (developed with 8 and 5 inputs) offer an exceptional ability to track solubility data of hydrocarbon gases with increasing pressure at low-temperature conditions compared to EOSs. Although the accuracy of EOSs has been lower than machine learning models, this does not mean questioning the capabilities of these thermodynamic equations. EOSs predict solubility data based on the thermodynamic variables within an analytical framework and they are valuable tools in the modeling of a wide range of industrial processes. Here, only a comparison between predictions of developed models and EOSs was made to clarify the high predictability of these models. Hence, machine learning models can be considered as an alternative to achieve accurate and fast predictions of the solubility of gases in brine in order to cover the disadvantages of EOSs mentioned earlier.

**Figure 8:**
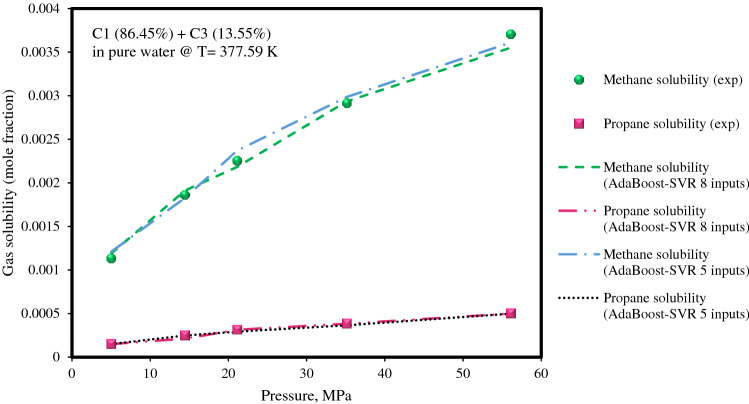
Experimental solubility data of methane and propane mixture in water at different operating pressures along with AdaBoost-SVR models predictions.

Next, the solubilities of methane and propane mixtures in pure water, which has been experimentally investigated by Amirijafari^23^ at a temperature of 377.59 K under high-pressure conditions, was predicted by the AdaBoost-SVR models, as demonstrated in Fig. 8. As depicted in the figure, both AdaBoost-SVR models correctly predicted the solubilities of methane and propane in pure water by increasing the pressure as an important parameter affecting solubility.

**Figure 9:**
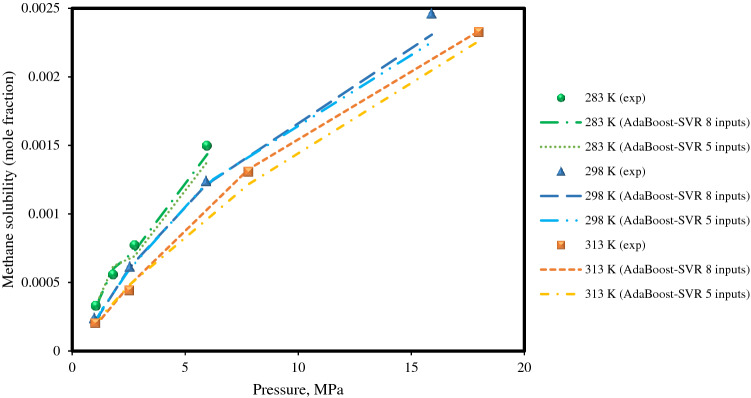
Experimental methane solubility data and AdaBoost-SVR models predictions for the methane + pure water system at different temperatures.

In the next step, the solubility of methane in water versus pressure at different temperatures was predicted by the AdaBoost-SVR models, which has been examined in the literature^9^. The solubilities of methane, as the basic constituent of natural gas, in pure water and aqueous electrolyte systems at different pressure and temperature is crucial for the petroleum industry. As shown in Fig. 9, the solubility of methane in water at various pressure and temperature conditions is accurately predicted by the AdaBoost-SVR models. As can be seen, the temperature has a decreasing impact on the methane’ solubility in water at the studied pressures, which is correctly estimated by the AdaBoost-SVR models.

**Figure 10:**
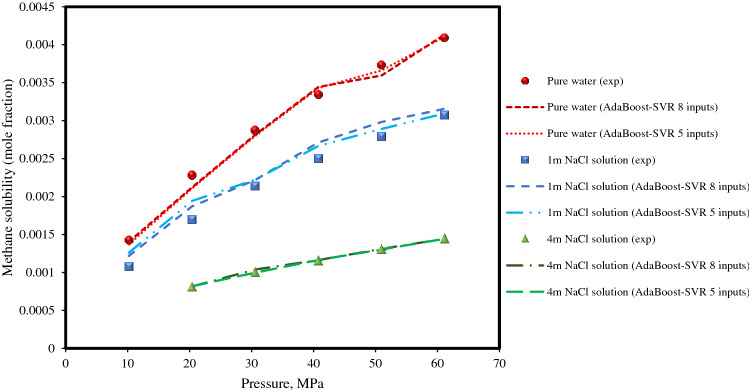
Experimental methane solubilities in water and aqueous NaCl solutions at a temperature of 324.65 K along with AdaBoost-SVR models predictions.

Eventually, the solubilities of methane in pure water and in aqueous NaCl solutions with different salt concentrations at a temperature of 324.65 K, which has been studied experimentally in the literature^67^, was predicted by the AdaBoost-SVR models. As can be observed in Fig. 10, the solubility of methane has an appreciable decrease with an increase in salt concentration or ionic strength of the solution. Again, both AdaBoost-SVR models provide accurate predictions for the systems of methane + water and methane + aqueous salt solution with different concentrations at different pressures with very little deviation from the experimental data.

### Sensitivity analysis

**Figure 11:**
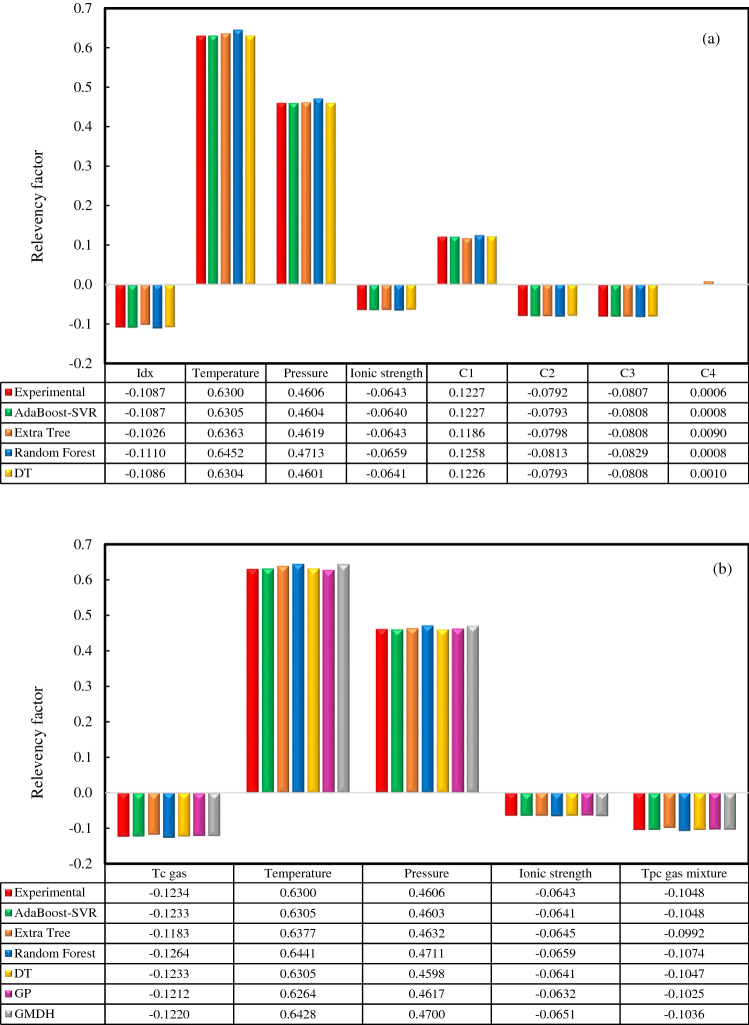
The impact of input variables on hydrocarbon gases solubility in water and aqueous electrolyte systems in the (**a**) first and (**b**) second modeling approaches.

In parametric studies, identifying the impacts of all inputs on the output can be valuable. As stated earlier, two modeling approaches with 8 and 5 inputs were adopted in this work. The first approach was that there were 8 inputs including the temperature, pressure, ionic strength of the solution, the mole percent of each component (C1, C2, C3, and C4) in the gas mixture, and carbon number (IDX) of the gas component whose solubility is to be predicted. On the other hand, the second approach considered 5 inputs containing the temperature, pressure, ionic strength of the solution, the pseudo-critical temperature of the gas mixture, and the critical temperature of the gas component whose solubility is to be predicted. To check the relative effects of these input variables on the solubilities of hydrocarbon gases in water and aqueous electrolyte systems, the relevancy factor (*r*)^98^ was employed in this research. It should be mentioned that the outcomes of all developed models and correlations developed in this work along with experimental data have been utilized for sensitivity analysis to make a comparison between the results of all models in both modeling approaches. Positive or negative values of *r* for an input parameter indicate a direct or inverse relationship between that parameter and the output, respectively. The higher value of *r* between an input variable and output, the greater the impact of that input on the solubilities of hydrocarbon gases in water and aqueous electrolyte systems. The subsequent equation is utilized for calculating the *r*-values for the input parameters^99^:

18 $$ r(inp_{i} ,\eta ) = \frac{{\sum\limits_{j = 1}^{n} {\left( {inp_{i,j} - inp_{m,i} } \right)\left( {\eta_{j} - \eta_{m} } \right)} }}{{\left( {\sum\limits_{j = 1}^{n} {\left( {inp_{i,j} - inp_{m,i} } \right)^{2} \sum\limits_{j = 1}^{n} {\left( {\eta_{j} - \eta_{m} } \right)^{2} } } } \right)^{0.5} }} $$

where *i* could be any of the input parameters considered for modeling; *inp*_*m,i*_ and *inp*_*i,j*_ respectively indicate the mean and *j*th value of the *i*th input parameter. *η*_*m*_ stands for the mean of predicted solubility of hydrocarbon gases in water and aqueous electrolyte systems and *η*_*j*_ is the *j*th value of predicted solubilities of hydrocarbon gases. Figure 11 illustrates the relative impacts of considered input variables on the solubilities of hydrocarbon gases in water and brine solutions. As seen in Fig. 11a, in the first modeling approach, the temperature, pressure, and methane (mole %) in the gas mixture had the greatest effects on hydrocarbon gases solubility. Also, the mole percent of the *n*-butane in the gas mixture was the least effective parameter for estimating the solubilities of hydrocarbon gases. Based on results, the temperature, pressure, and mole percent of methane and *n*-butane in the gas mixture have direct effects, and mole percent of ethane and propane in the gas mixture, IDX, and ionic strength of the solutions have reverse effects on the solubility of investigated hydrocarbon gas. An increase in the ionic strength of the solution decreases the solubilities of hydrocarbon gases in aqueous electrolyte systems. In the second modeling approach, as shown in Fig. 11b, the results of sensitivity analysis for temperature, pressure, and ionic strength variables have been obtained quite similarly to the previous case. Moreover, the pseudo-critical temperature of the gas mixture and the critical temperature of the gas components have negative effects on the solubility of light hydrocarbon gases and their mixture in brine, which exhibits that the solubility decreases with the rise of these parameters. As inferred from the results of the sensitivity analysis of both modeling approaches, the feature-solubility correlations are completely independent of machine learning frameworks and the impact of each specific input variable applied for modeling in each model or correlation developed in this work are the same and similar to the laboratory results.

### Implementation of Leverage method

Finally, the degree of precision of utilized data along with the application scope of the AdaBoost-SVR models was examined using the Leverage approach^100–102^, which can assess the validity of these model and solubility databank. The subsequent equation was utilized to calculate the variations of the prognosticated solubility values by the model from the real data, which is named standardized residuals (*R*)^103^:

19 $$ R_{z} = \frac{{e_{z} }}{{\left( {MSE\left( {1 - H_{zz} } \right)} \right)^{0.5} }} $$

in which, the mean square error of the predictive tool is shown by *MSE*; *H*_*zz*_ shows Leverage of the *z*th data; and *e*_*z*_ denotes the variation of the estimations from the experiments of the *z*th data. Afterward, the following formula is utilized to calculate the values of Hat matrix Leverage^104^:

20 $$ {\text{H = K (K}}^{{\text{T}}} {\text{K)}}^{{ - 1}} {\text{K}}^{{\text{T}}} $$

where *K*^*T*^ shows the transpose of the matrix *K,* which is (*g* × *c*) matrix; *g* and *c* indicate the number of databank points and the number of input variables, respectively. Besides, the critical Leverage limit (H^*^) is achieved using *3*(*c* + *1*)/*g*.

**Figure 12:**
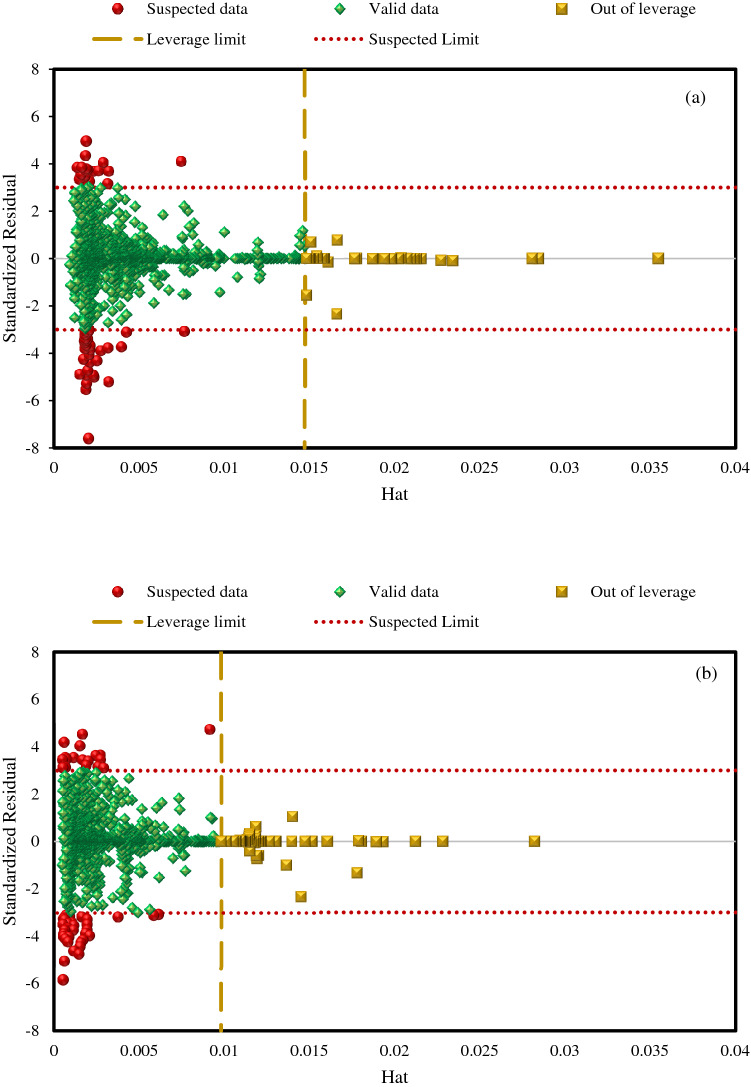
Detection of applicability area, suspected data, and outliers of AdaBoost-SVR models developed with (**a**) 8 inputs and (**b**) 5 inputs.

The reliable zone is considered to be the cut-off area of *R*-values (−3 and 3) and *H*_*zz*_ ≤ H*, as shown in William's plot in Fig. 12. This figure exhibits that the bulk of data, called valid data, rested in the reliable zone that proves the high reliability of the hydrocarbon solubility databank and high validation of the AdaBoost-SVR models. For the AdaBoost-SVR model developed with 8 inputs, as depicted in Fig. 12a, quantitative identification of the outliers of the used databank shows that only 54 data points (2.94% of the whole data) have an *R*-value outside the range of −3 to 3, which is considered suspected data. In addition, only 35 data points (1.91% of the whole data) have *H*_*zz*_ > 0.0147, which is regarded as out of Leverage data, while other data have acceptable Leverage (*H*_*zz*_ ≤ 0.0147). For the AdaBoost-SVR model developed with five inputs, due to the reduction of the number of input variables, the critical Leverage limit value is reduced to H^*^ = 0.0098*,* and the application scope of the model becomes more limited. However, there is no specific change in the number of suspected data points (54 data points means 2.94% of the whole data), and only the out of Leverage data has increased to 70 (3.81% of the whole data). As shown in Fig. 12b, these points are also predicted by the model with a very low error, and they are just statistically beyond the critical Leverage limit. Hence, it cannot be considered a negative point for the model. The results of the Leverage mathematical method reveal the validity of the hydrocarbon solubility databank and the high credit of both AdaBoost-SVR models in estimating the solubility of hydrocarbon gases in water and brine solution systems.

## Conclusions

In the present study, the solubilities of the principal hydrocarbon components of natural gas in water and aqueous electrolyte solutions were modeled utilizing six machine learning algorithms. A large databank (1836 experimental data points) of hydrocarbon gases solubility was gathered from numerous sources of literature to cover a wide range of temperature and pressure conditions. Two different approaches including eight and five inputs were adopted for modeling. Also, three famous EOSs, including PR, VPT, and SRK were used in comparison with machine learning models. Based on graphical and statistical analyses, the best-developed models in this work, namely AdaBoost-SVR developed with eight and five inputs, are able to predict the solubility of hydrocarbon gases and their mixture with an overall AAPRE of 10.65% and 12.02%, respectively, and R^2^ of 0.9999. The AdaBoost-SVR models outperform other models developed in this work, EOSs, and intelligence models proposed in the literature. Also, the Random Forest, DT, and Extra Tree models are positioned subsequent to the AdaBoost-SVR model in terms of high precision in predicting test collection in both modeling approaches. Despite higher errors than machine learning models, two mathematical correlations generated by the GMDH and GP techniques had satisfactory outcomes. Among the EOSs, VPT, SRK, and PR are ranked in terms of good predictions, respectively. Based on sensitivity analysis, the temperature and pressure had the greatest effect on hydrocarbon gases solubility in both modeling approaches. Regarding the gas mixture composition (C1–C4), the percentage of methane and *n*-butane in the gas mixture was the most and least effective parameter for predicting the solubility of hydrocarbon gases in brine, respectively. Additionally, an increase in the ionic strength of the solution and the pseudo-critical temperature of the gas mixture decreases the solubilities of hydrocarbon gases in aqueous electrolyte systems. Moreover, the influence of input variables on light hydrocarbon gases solubility is completely independent of machine learning frameworks. Eventually, the investigation of the Leverage technique proved the high validity of the hydrocarbon solubility databank and the high credit of the AdaBoost-SVR models in predicting hydrocarbon gases solubility in water and aqueous electrolyte systems.

## Supplementary Information

Supplementary Information.

## Data Availability

All the data have been collected from literature. We cited all the references of the data in the manuscript. However, the data will be available from the corresponding author on reasonable request.

## Abbreviations

AAPRE: Average absolute percent relative error
AdaBoost: Adaptive boosting
AdaBoost-SVR: Adaptive boosting support vector regression
DT: Decision tree
EOS: Equation of state
ET: Extra tree
exp: Experimental
PR: Peng–Robinson
pred: Predicted
RMSE: Root mean square error
r: Relevancy factor
SD: Standard deviation
SVR: Support vector regression
SRK: Soave–Redlich–Kwong
RF: Random forest
R^2^: Coefficient of determination
VPT: Valderrama modification of the Patel–Teja
