# Supplementary material for: Modeling the solubility of light hydrocarbon gases and their mixture in brine with machine learning and equations of state
Source: Sci Rep. 2022 Sep 2;12:14943. doi: 10.1038/s41598-022-18983-2 (PMC9440136; doi:10.1038/s41598-022-18983-2)
Supplement: Supplementary file 1 — Supplementary Information. [file 41598_2022_18983_MOESM1_ESM.docx]

**Modeling the solubility of light hydrocarbon gases and their mixture in brine with machine learning and equations of state**

Mohammad-Reza Mohammadi ^1^, Fahimeh Hadavimoghaddam ^2,3^, Saeid Atashrouz ^4,^ *, Ali Abedi ^5^, Abdolhossein Hemmati-Sarapardeh 1, ^6^, *, Ahmad Mohaddespour ^7,^ *

*^1^ Department of Petroleum Engineering, Shahid Bahonar University of Kerman, Kerman, Iran*

*^2^ Key Laboratory of continental shale hydrocarbon accumulation and efficient development (Northeast Petroleum University), Ministry of Education, Northeast Petroleum University, Heilongjiang, Daqing 163318, China*

*^3^ Institute of Unconventional Oil & Gas, Northeast Petroleum University, Daqing, 163318, China*

*^4^ Department of Chemical Engineering, Amirkabir University of Technology (Tehran Polytechnic), Tehran, Iran*

*^5^ College of Engineering and Technology, American University of the Middle East, Kuwait*

*^6^ College of Construction Engineering, Jilin University, Changchun, China*

*^7^ Department of Chemical Engineering, McGill University, Montreal, QC H3A 0C5, Canada*

**Supplementary file:**

**Table S1.** The formulas of EOSs.

| Reference | PVT relation | EOS |
| --- | --- | --- |
| ^1,2^ | $P=\frac{RT}{\nu-b}-\frac{a_{c}\alpha}{\nu(\nu+b)}$ | SRK |
| ^1,2^ | $P=\frac{RT}{\nu-b}-\frac{\alpha}{\nu(\nu+b)+b\left(\nu-b\right)}$ | PR |
| ^3-5^ | $P=\frac{RT}{\nu-b}-\frac{a(T)}{\nu(\nu+b)+c\left(\nu-b\right)}$ | VPT |

**Table S2.** The parameters of EOSs.

| Reference | Parameters | EOS |
| --- | --- | --- |
| ^1,2^ | $\begin{matrix} a_{C}=0.42747\frac{R^{2}T_{C}^{2}}{P_{C}} \\ b=0.08664\frac{RT_{C}}{P_{C}} \\ m=0.480+1.574\omega-0.176\omega^{2} \\ \alpha=\left[1+m\left(1-\sqrt{T_{r}}\right)\right]^{2} \end{matrix}$ | SRK |
| ^1,2^ | $\begin{matrix} \alpha=a_{C}\alpha(T) \\ \alpha(T)=\left[1+m\left(1-\sqrt{T_{r}}\right)\right]^{2} \\ a_{C}=0.4572\frac{(RT_{C})^{2}}{P_{C}} \\ m=0.3746+1.5422\omega-0.26992\omega^{2} \\ b=0.07780\frac{RT_{C}}{P_{C}} \end{matrix}$ | PR |
| ^3-5^ |  | VPT |

**Table S3.** Acentric factors and critical properties of the light hydrocarbon gases and water ^5-7^.

| ω | T_c_ (K) | P_c_ (MPa) | Z_c_ | Substance |
| --- | --- | --- | --- | --- |
| 0.0108 | 190.58 | 4.604 | 0.288 | Methane |
| 0.09896 | 305.42 | 4.879 | 0.285 | Ethane |
| 0.15176 | 369.82 | 4.249 | 0.281 | Propane |
| 0.1931 | 425.18 | 3.797 | 0.274 | n-Butane |
| 0.3449 | 647.13 | 22.055 | 0.2294 | Water |


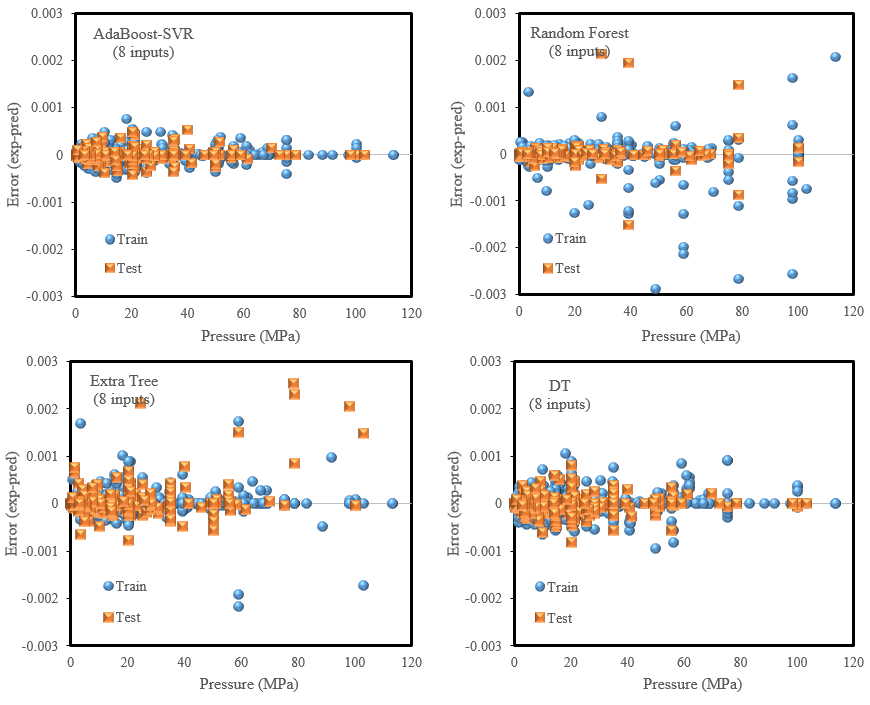


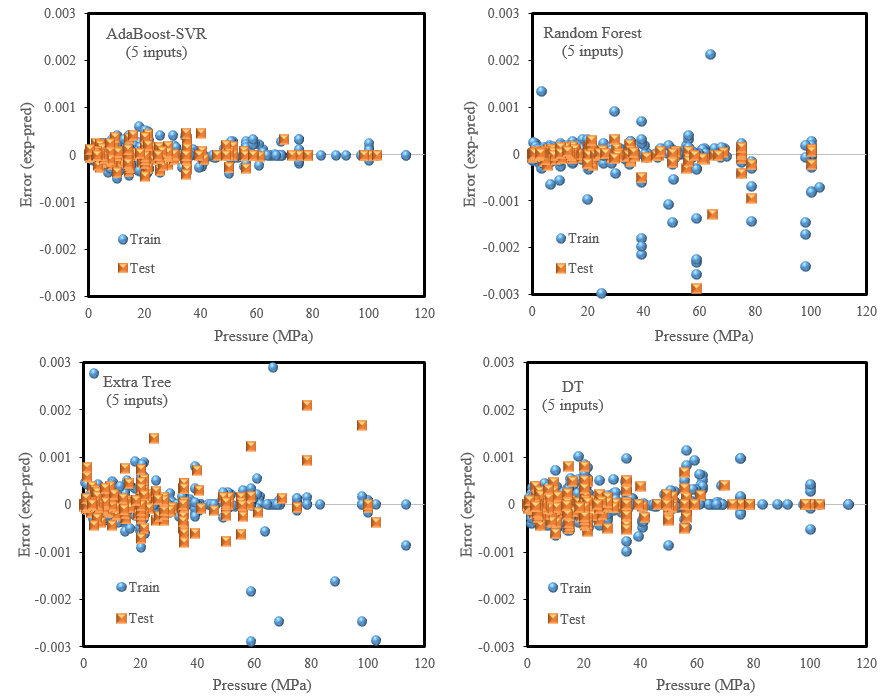


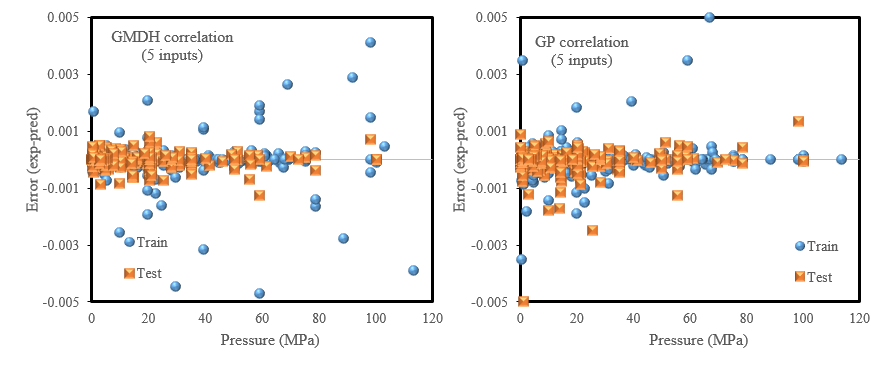


1. based on pressure


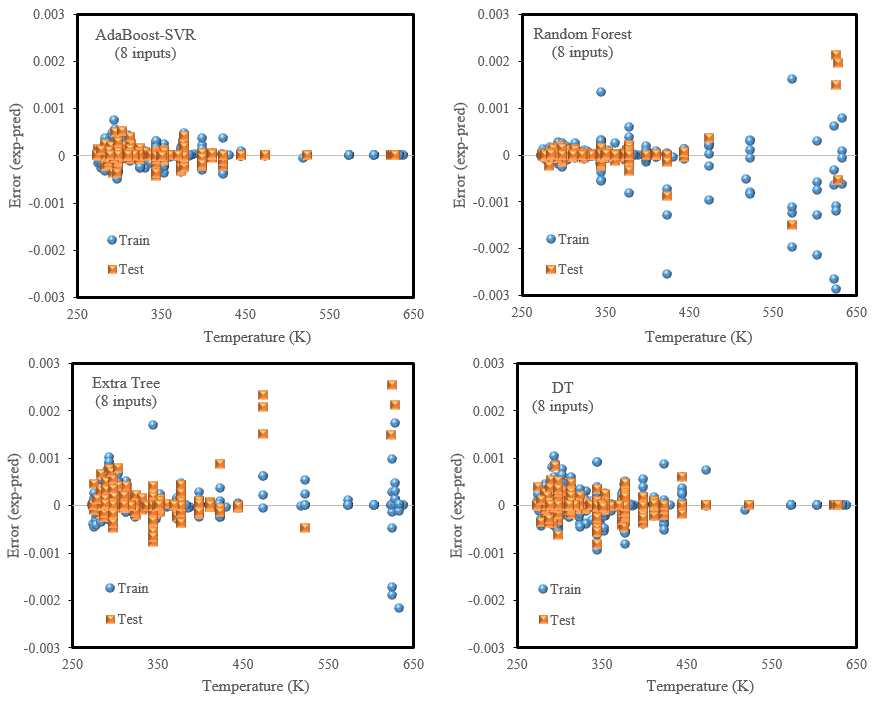


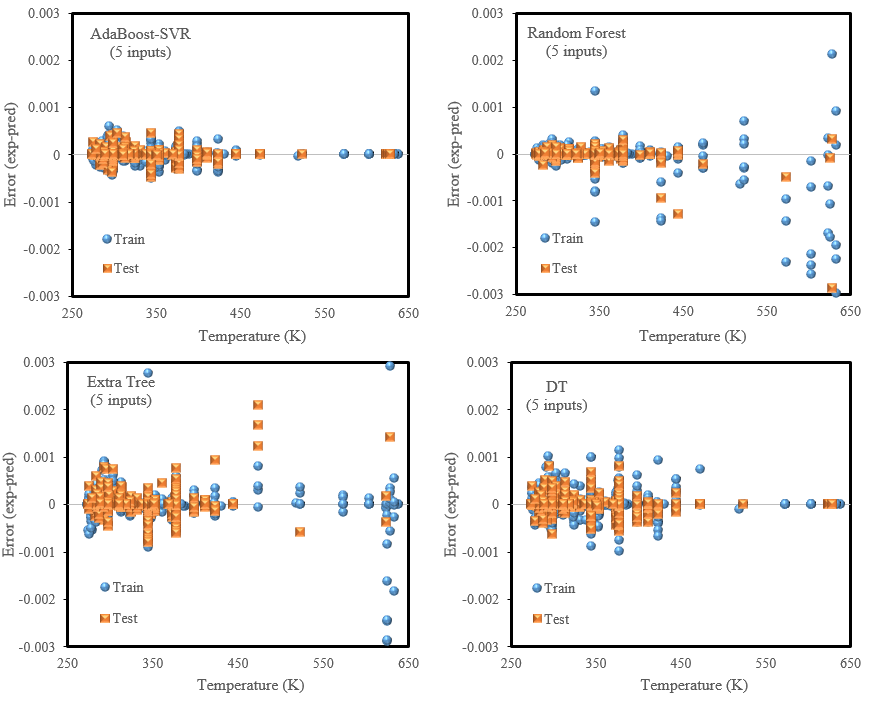


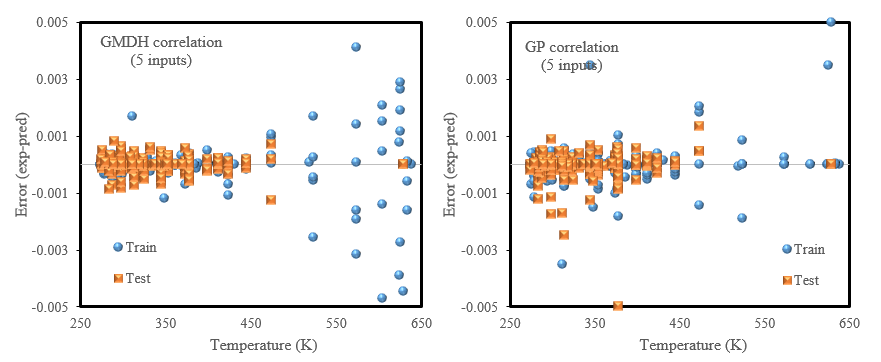


1. based on temperature

**Fig. S1.** Error distribution graphs of the developed machine learning models and correlations.

**References**

1 Pedersen, K. S., Christensen, P. L. & Shaikh, J. A. *Phase behavior of petroleum reservoir fluids*. (CRC press, 2014).

2 Péneloux, A., Rauzy, E. & Fréze, R. A consistent correction for Redlich-Kwong-Soave volumes. *Fluid phase equilibria* **8**, 7-23 (1982).

3 Valderrama, J. O. A generalized Patel-Teja equation of state for polar and nonpolar fluids and their mixtures. *Journal of chemical engineering of Japan* **23**, 87-91 (1990).

4 Avlonitis, D., Danesh, A. & Todd, A. Prediction of VL and VLL equilibria of mixtures containing petroleum reservoir fluids and methanol with a cubic EoS. *Fluid Phase Equilibria* **94**, 181-216 (1994).

5 Danesh, A. *PVT and phase behaviour of petroleum reservoir fluids*. (Elsevier, 1998).

6 Chapoy, A. *et al.* Solubility measurement and modeling for the system propane–water from 277.62 to 368.16 K. *Fluid Phase Equilibria* **226**, 213-220 (2004).

7 Chapoy, A., Mohammadi, A. H., Richon, D. & Tohidi, B. Gas solubility measurement and modeling for methane–water and methane–ethane–n-butane–water systems at low temperature conditions. *Fluid phase equilibria* **220**, 113-121 (2004).
